# The *N*6‐methyladenosine RNA landscape in the aged mouse hippocampus

**DOI:** 10.1111/acel.13755

**Published:** 2022-12-09

**Authors:** He Huang, Renhua Song, Justin J.‐L. Wong, Victor Anggono, Jocelyn Widagdo

**Affiliations:** ^1^ Clem Jones Centre for Ageing Dementia Research Queensland Brain Institute, The University of Queensland Brisbane Queensland Australia; ^2^ Epigenetics and RNA Biology Program Centenary Institute The University of Sydney Camperdown New South Wales Australia; ^3^ The University of Sydney Faculty of Medicine and Health Camperdown New South Wales Australia

**Keywords:** aged brain, epitranscriptomic, hippocampus, *N*6‐methyladenosine, RNA methylation

## Abstract

The aged brain is associated with an inevitable decline in cognitive function and increased vulnerability to neurodegenerative disorders. Multiple molecular hallmarks have been associated with the aging nervous system through transcriptomics and proteomic studies. Recently, epitranscriptomic analysis has highlighted the role of RNA chemical modification in various biological processes. In particular, *N*6‐methyladenosine (m6A), the most abundant internal modification in eukaryotic mRNAs, has been functionally linked to multiple aspects of RNA metabolism with the roles of m6A in processes such as learning and memory, leading to our current investigation of how the m6A‐transcriptomic landscape is shaped during aging. Using the inbred C57BL/6 line, we compared the m6A‐transcriptomic profiles from the hippocampi of young (3‐month‐old) and aged (20‐month‐old) mice. Methylated RNA immunoprecipitation (MeRIP)‐sequencing analysis revealed hyper‐ and hypomethylation in 426 and 102 genes, respectively, in the aged hippocampus (fold change >1.5, false discovery rate <0.05). By correlating the methylation changes to their steady‐state transcript levels in the RNA‐Seq data, we found a significant concordance between m6A and transcript levels in both directions. Notably, the myelin regulator gene *Gpr17* was downregulated in the aged hippocampus concomitant with reduced m6A levels in its 3'UTR. Using reporter constructs and mutagenesis analysis, we demonstrated that the putative m6A sites in the 3'UTR of *Gpr17* are important for mRNA translation but not for regulating transcript stability. Overall, the positive correlation between m6A and the transcript expression levels indicates a co‐transcriptional regulation of m6A with gene expression changes that occur in the aged mouse hippocampus.

## Graphical abstract

The RNA modification N6‐methyladenosine (m6A) regulates many aspects of RNA metabolism. Transcriptomic profiling reveals that the changes in m6A in the aged mouse hippocampus correlate with the overall changes in transcript levels. Our study indicates a co‐transcriptional regulation of m6A with age‐mediated gene expression changes in the mouse hippocampus.

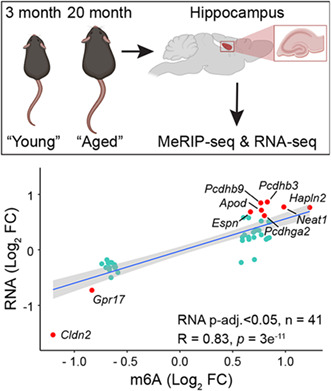

## 1 INTRODUCTION, RESULTS, AND DISCUSSION

Over 170 different RNA modifications have been identified so far (Cantara et al., 2011), among which m6A is the most frequently found internal modification in eukaryotic messenger RNAs (mRNAs) (Dominissini et al., 2012; Meyer et al., 2012). The molecular actions of m6A have been associated with various aspects of mRNA regulation in a myriad of biological systems, including the central nervous system (Livneh et al., 2020; Widagdo & Anggono, 2018; Widagdo, Wong, & Anggono, 2022). Depletion or modulation of m6A has led to its implication in multiple physiological functions of the mammalian brain, including the regulation of cortical development (Yoon et al., 2017), learning and memory (Koranda et al., 2018; Shi et al., 2018; Walters et al., 2017; Widagdo et al., 2016; Zhang et al., 2018), and the stress response (Engel et al., 2018). Recent studies have also linked m6A dysregulation to the pathology of age‐related neurodegenerative disorders such as Alzheimer's disease (Huang et al., 2020; Shafik et al., 2021; Zhao et al., 2021), raising the question of how the m6A‐transcriptomic landscape is regulated in the brain during normal aging.

The hippocampus is particularly vulnerable to age‐related decline, including in functions such as learning and memory, and has been associated with changes in transcriptional, epigenetic, and homeostatic cellular processes during aging (Fan et al., 2017). To examine the m6A‐RNA regulation in neurocognitive aging, we used a well‐established C57BL/6 mouse model of age‐related cognitive decline (Bach et al., 1999; Verbitsky et al., 2004), to compare the m6A‐transcriptomic landscape of young (3‐month‐old) and aged (20‐month‐old) mice (Figure S1a). Hippocampal RNAs were first examined for the modulation of several of known aging‐related genes. In line with previous reports (Bettio et al., 2017; Cribbs et al., 2012; Stilling et al., 2014), our quantitative reverse transcription PCR (qRT‐PCR) analyses detected an upregulation of *Neat1* long non‐coding RNA, the complement factor *C4b*, and the neuroinflammatory gene *Trem2*, as well as the downregulation of *Cldn2*, a tight junction component, in the aged samples (Figure S1b). RNA sequencing (RNA‐Seq) analysis of the hippocampi (three biological replicates per age group) revealed a total of 125 significantly upregulated and 121 downregulated genes (fold changes ≥1.5) in the aged mice (Figure S1c,d). Gene ontology (GO) analysis of differentially expressed genes highlighted the enrichment of biological processes previously associated with aging, including upregulation in the immune and defiance response pathways (*C3*, *Il33*, and *Trem2*) and downregulation in extracellular matrix organization (*Sulf1*, *Col9a3*, *Col8a2*, and *Col3a1*) (Figure S1e).

**FIGURE 1:**
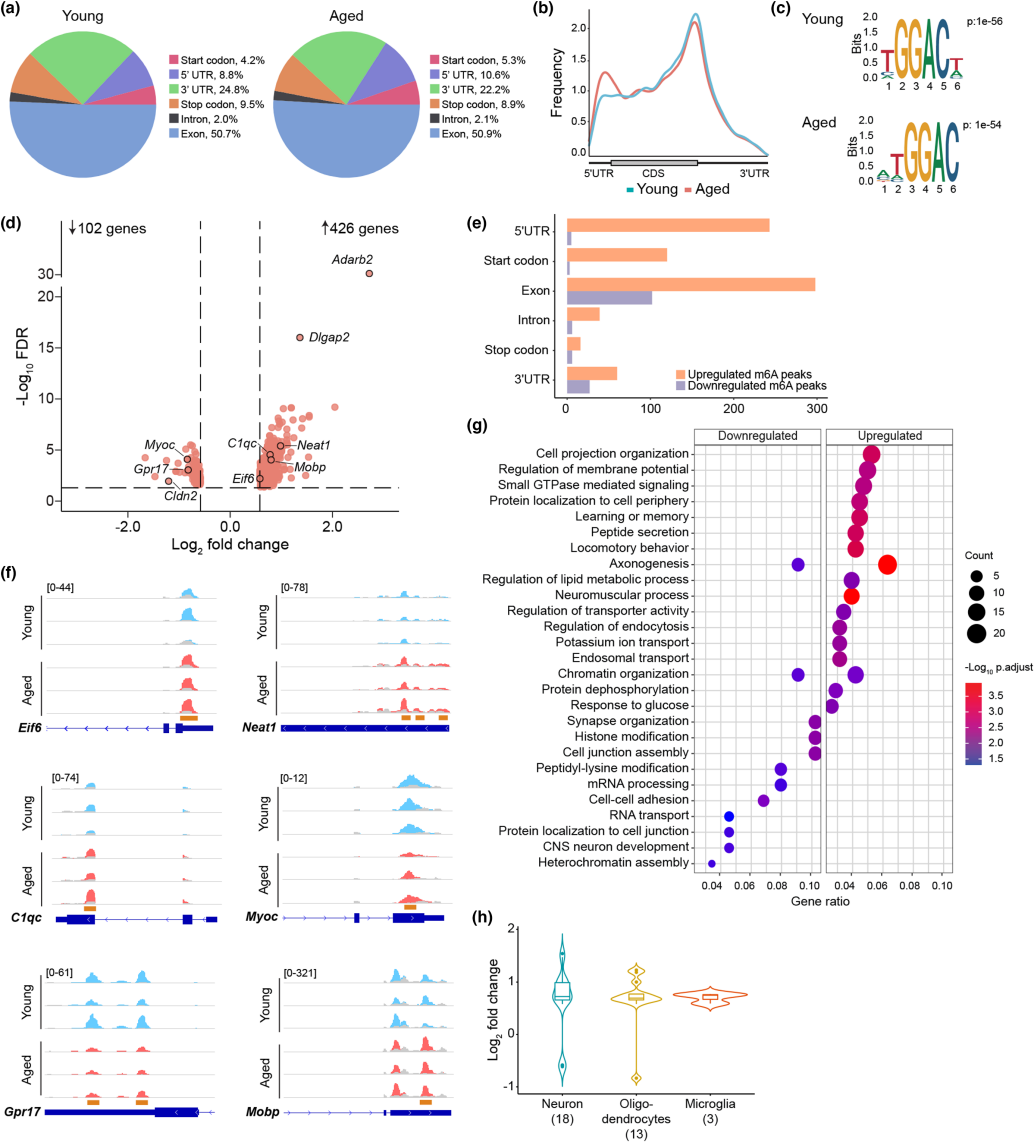
Aged hippocampal m6A‐transcriptomic profiles. (a and b) Distribution and metaplot of all MeRIP‐Seq‐identified peaks in the hippocampal RNAs of young and aged mice. (c) The enriched consensus motif associated with m6A peaks in young and aged mice. (d) Volcano plot showing aged‐associated increases and decreases in methylation (fold change >1.5; FDR >0.05). (e) Location of the up‐ and downregulated m6A peaks in mRNAs. (f) Representative traces of up‐ and down‐m6A‐regulated transcripts in the aged hippocampus. Blue and red traces are immunoprecipitated coverages of young and aged samples, respectively, overlaid with the input (gray) coverages. Orange bars indicate the location of DM peaks. (g) GO enrichment analyses of age‐modulated m6A transcripts. (h) Cell type‐specific analyses of DM genes based on Guo et al. (2019) showing a general hypermethylation in 18 neuron‐, 13 oligodendrocyte‐, and three microglia‐specific genes.

We next performed methylated RNA immunoprecipitation sequencing (MeRIP‐Seq) analysis by coupling the polyA‐enriched RNA to m6A‐immunoprecipitation protocol (Widagdo et al., 2016). A total of 8084 and 7527 m6A peaks were detected in the young and aged hippocampus, respectively, which corresponded to 4834 and 4585 genes (>92% of which were protein‐coding) (Figure S2a). A similar distribution along the mRNAs was observed in young and aged mice, with the highest frequencies located in the exons and 3′ untranslated regions (3'UTR), particularly in the vicinity of the stop codon (Figure 1a,b). Sequence motif analyses of the peaks also revealed an enrichment of the core m6A consensus motif GGAC in both groups (Figure 1c).

Differential analysis of the m6A peaks revealed a total of 528 differentially methylated (DM) genes (862 peaks; fold change >1.5 and FDR <0.05) in the aged compared with the young mice. Hypermethylation in 426 genes was mostly found in their coding sequence (exonic) and 5'UTR regions, whereas hypomethylation in 102 genes mostly resided in the coding sequence (exonic) and 3'UTR regions (Figure 1d,e; Table S1). Examples of DM genes in the aged animals included *Eif6*, *C1qc*, *Neat1*, and *Mobp* with hypermethylated peaks, and *Gpr17* and *MyoC* with hypomethylated peaks (Figure 1f). Overall, the top biological processes associated with hypermethylated genes were the cell projection organization, regulation of membrane potential, and small GTPase signal transduction (Rap1 signaling), relevant to functions of diverse types of synapses (Figure 1g; Figure S2c; Table S2). By contrast, the top biological processes associated with hypomethylated genes were synapse organization, histone modification, and cell junction assembly (Figure 1g). Interestingly, both hyper‐ and hypomethylation of distinct genes were associated with axonogenesis and chromatin organization (Figure S2b), highlighting a multidimensional role of m6A in regulating specific molecular processes during aging (Figure S2b).

**FIGURE 2:**
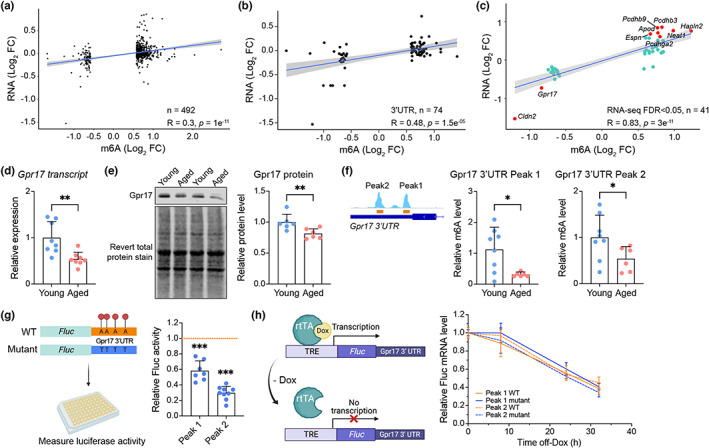
Concordant changes in m6A and transcript levels in aged hippocampus. (a) Regression analysis (Spearman's correlation) of DM genes showing the relationship between m6A fold change and its RNA transcript level in aged animals. Similar analyses were applied to (b) a subset of DM genes with m6A changes in the 3'UTR and (c) a subset of genes co‐detected as differentially expressed genes from the RNA‐Seq datasets (FDR <0.05 regardless of fold change). Turquoise dots denote genes with significant expression changes in the aged hippocampus (FDR <0.05, no fold change applied, 41 genes), and red dots denote genes with significant differential fold change >1.5 (9 genes). (d) qRT‐PCR analysis of *Gpr17* normalized to *Rpl13a* (e) Representative Western blot showing Gpr17 protein levels in the young and aged mouse hippocampus and the total stain by Revert. Quantification of Gpr17 protein levels normalized to the Revert staining is shown in the bar graph (*n* = 6). (f) MeRIP‐qPCR validation of m6A changes in peak 1 and peak 2 of *Gpr17*. (g) Measurement of the luciferase signals in cells transfected with Fluc‐Gpr17(3'UTR) reporter constructs showing the relative luciferase expression in the mutant peak 1 or peak 2 transfection relative to the corresponding wild‐type levels. (h) Schematics of the tetracycline‐inducible reporter constructs. The presence of doxycycline (Dox) binds the rtTA transcription factor and subsequently binds to the response element and induces the luciferase expression. In the absence of Dox, the rtTA does not bind the response element and the expression of luciferase is inhibited. The measurement analysis reveals the levels of remaining luciferase mRNA normalized to the β‐actin gene in the cells. All data are presented as mean ± *SD* (Mann–Whitney *U* test, *p < 0.05, **p < 0.01, ***p < 0.0001).

As one of the functions of m6A is to modulate transcript stability (Wang, Li, et al., 2014; Wang, Lu, et al., 2014), we examined the correlation between changes in m6A and their transcript levels in aging. Our analysis revealed that DM genes displayed an overall modest but significant linear correlation with the RNA expression changes during aging (*R* = 0.3, *p* = 1 e^−11^; Figure 2a). Based on the location of the peaks, DM genes associated with 3'UTR‐m6A peaks exhibited the highest linear correlation (74 genes, *R* = 0.48, *p* = 1.5 e^−5^; Figure 2b). Applying the regression analysis on a subset of DM genes with differential transcript levels based on our RNA‐Seq analysis (FDR <0.05) produced a stronger linear correlation (41 genes, *R* = 0.83, *p* = 3 e^−11^; Figure 2c). The genes with the most significant co‐regulated m6A‐transcript changes included the upregulated *Pcdh9*, *Pcdh3*, *Hapln2*, *Apod*, *Neat1*, *Espn*, and *Pcdhga2* and the downregulated *Gpr17* and *Cldn2* in the aged hippocampus.

One of the top m6A co‐regulated transcripts was downregulated in its overall transcript and m6A levels, *Gpr17,* has recently emerged as a key regulator of myelin in the aging brain (Marschallinger et al., 2015; Rivera et al., 2021). As two distinct peaks at the 3'UTR of *Gpr17* were significantly decreased in the aged hippocampus (Figure 1f), we further investigated a possible role of m6A in *Gpr17* transcript regulation. The downregulation of *Gpr17* at the transcript and protein levels in the aged hippocampus was first confirmed by qRT‐PCR and Western blot analyses (Figure 2d,e). Using MeRIP‐qPCR assays, we further verified the decreased levels of m6A in the proximal (“peak 1”) and distal (“peak 2”) 3'UTR of *Gpr17* (Figure 2f). To study the functional importance of the m6A‐targeted sites, we generated mini‐gene constructs consisting of the reporter firefly luciferase *Fluc* gene fused with the 3'UTR sequence of *Gpr17* containing either peak 1 or peak 2 (Figure 2g). When transfected in HEK293 cells, the transcript levels of *Fluc* measured by qPCR were comparable between wild‐type and mutant constructs (data not shown); however, mutations of either peak resulted in a significant reduction in the firefly luciferase levels compared with the wild‐type 3'UTR construct (Figure 2g). This result suggested a role of the 3'UTR m6A sites in the regulation of translation or transcript stability. To determine the transcript degradation rates associated with the 3'UTR sequences, we generated an inducible tetracycline‐controlled gene expression system to enable the induction of *Fluc* transcription only in the presence of doxycycline and measured the level of the remaining mRNA in a chase assay. Following doxycycline removal, the mRNA decay profiles associated with all reporter constructs were similar across groups (Figure 2h). These data suggest that m6A methylation in the 3'UTR of *Gpr17* mRNA regulates translation efficiency, but not transcript stability, at least in a reconstituted system. GPR17 is predominantly expressed in oligodendrocytes, the myelinating cells of the central nervous system, and its downregulation is required for active myelination (Chen et al., 2009). By contrast, upregulation of GPR17 is associated with demyelinating diseases (Lecca et al., 2020). We propose that modulation of Gpr17 translation by m6A may serve as a post‐transcriptional mechanism that dampens its function during aging.

Akin to epigenetic mechanisms, the role of RNA modification in modulating cellular changes associated with the aging process has been much anticipated (McMahon et al., 2021). However, despite the plethora of functions associated with m6A, few studies have explored the role of m6A in the context of aging. An overall reduction in m6A modifications has been reported in the peripheral blood mononuclear cells derived from aged relative to young individuals (Min et al., 2018). In the central nervous system, examination of the mouse cerebral cortex showed an overall hypermethylation in the moderate‐age (12‐month‐old) mice compared with young ones, and a similar trend was observed in the post‐mortem aged human brain compared with that of adolescents (Shafik et al., 2021). In our study, aged male mice also had higher proportion of hypermethylated mRNAs in their hippocampal m6A landscape, with the age‐related increase in m6A being attributable to many cell‐type‐specific genes in neurons (excitatory and inhibitory), oligodendrocytes, and microglia (Figure 1h). Although the sex‐specific molecular changes that have been reported in the hippocampus during aging are subtle (Berchtold et al., 2008), the possibility of sex differences in the m6A profile, such as those leading to differential neuroinflammation and microglial activation during aging (Mangold et al., 2017), cannot be discounted.

Our observation of a general concordant regulation of m6A and its transcript levels in the aged hippocampus may be explained by co‐transcriptional regulation of m6A (Aguiló et al., 2015; Huang et al., 2019). An increasing number of studies have reported a complex cross‐regulation between m6A RNA and the transcriptional machinery, whereby m6A may play an active role in modulating nascent RNA synthesis via chromatin‐associated factors (Lee et al., 2016; Li et al., 2020; Liu et al., 2020; Widagdo, Anggono, & Wong, 2022; Xu et al., 2022). We propose that dissecting the functional impact of m6A on transcription and vice versa, by manipulating m6A biogenesis in animal models, will be a critical future step. Although we did not detect changes in the expression of any m6A readers in the aged hippocampus (data not shown), a recent study has shown that the mislocalization of an indirect m6A reader HNRNPA2B1 and the associated m6A‐modified RNA is driven by tau pathology in Alzheimer's disease (Jiang et al., 2021). Therefore, investigating the state of m6A writers, erasers, and readers (localization, activity, and post‐translational modifications) will contribute toward understanding the role of m6A in regulating gene expression and its functional significance in the aging brain.

## 2 EXPERIMENTAL PROCEDURES

### 2.1 Animals

Male C57BL/6 mice, housed on a 12‐h light/12‐h dark schedule and fed ad libitum, were used for this study. At 12 weeks or 20 months of age, the mice were sacrificed by rapid decapitation. Hippocampal tissues were rapidly dissected and flash‐frozen in liquid nitrogen. Experiments were approved by the University of Queensland Animal Ethics Committee (AEC approval number QBI/047/18).

### 2.2 RNA isolation and sequencing

Total RNA extraction was performed from individual hippocampal tissue using NucleoZol and NucleoSpin RNA Set (Macherey‐Nagel) with rDNase treatment. The RNA quality, all with RIN >8.7, was verified using the Agilent 2100 Bioanalyzer (Agilent Technologies). Three biological replicates per age group were selected from the pre‐validation qPCR assay and subjected to further poly(A)‐RNA enrichment using Oligo d(T)_25_ magnetic beads (NEB). Approximately 1.4 μg Poly(A)‐RNA was chemically fragmented to ~100 nt using NEBNext RNA fragmentation buffer (NEB). RNA immunoprecipitation with m6A‐specific antibody (Synaptic System; cat no. 202003) was performed as described previously (Widagdo et al., 2016). The input and immunoprecipitated RNA were converted to libraries using the SMARTer Stranded Total RNA‐Seq Kit v2 Pico Input Mammalian (Takara Bio) and their dual combinatorial indices for multiplexing. For RNA sequencing, libraries were generated using the NEBNext Ultra Directional RNA Library Prep Kit for Illumina (NEB). All libraries were sequenced on an Illumina HiSeq 4000 for paired‐end 150‐bp sequencing (Novogene).

### 2.3 MeRIP‐Seq and RNA‐Seq analyses

Detailed protocols to analyze the MeRIP‐Seq and RNA‐Seq data are described in Appendix S1 (Extended Methods).

### 2.4 Quantitative PCR

Reverse transcription of total (gene expression) or input and m6A‐immunoprecipitated RNA (for MeRIP‐qPCR) was performed using SuperScript IV (Invitrogen). The expression levels of candidate genes or region‐specific m6A were analyzed using SYBR‐green amplification with specific primers (Appendix S1) on the Rotor‐gene (Qiagen).

### 2.5 Western blotting

To determine the protein levels of Gpr17, hippocampal tissue was homogenized in cold lysis buffer (50 mM HEPES pH 7.4, 2 mM EDTA, 2 mM EGTA, 2 mM PMSF, complete protease inhibitor EDTA free and phosphatase inhibitor), followed by the addition of 2% SDS. The lysates were subjected to Western blotting as described previously (Zhu et al., 2017). The membrane was probed with specific antibody against Gpr17 (sc‐514,723, Santa Cruz). Protein was stained using Revert total protein stain (LI‐COR), and band intensities were quantified using Image Studio (LI‐COR).

### 2.6 Luciferase assay

Mini gene reporter constructs containing firefly luciferase fused with the 3'UTR of Gpr17 (Appendix S1) were transfected in HEK293 cells together with the control pRL‐TK plasmid (Promega) using Lipofectamine 2000 (Invitrogen). Cell lysates were collected 48 h later to measure the luciferase activity (Firefly/Renilla) using the Dual‐Luciferase Assay Kit (PerkinElmer).

### 2.7 mRNA stability assay

Tetracycline‐inducible *Tet‐Gpr17* plasmids containing luciferase‐Gpr17‐3'UTR (Appendix S1) were transfected in HEK293 cells together with pCAG‐TetON‐3G (Addgene, plasmid #96963). Doxycycline (final concentration 1 μg/ml) was added overnight to induce luciferase expression. Following doxycycline removal by complete medium change, RNA was isolated at 0, 8, 24, and 30 h for qPCR analysis of *luciferase* transcript levels.

### 2.8 Statistical analysis

Statistical analyses were conducted using the R package or Prism (GraphPad). Data from two groups were analyzed using a two‐tailed Mann–Whitney U test, and all error bars were expressed as standard deviation (*SD*) of the mean. *p*‐values are indicated in the figures and figure legends.

## AUTHOR CONTRIBUTIONS

JW conceived the project. JW and VA supervised the study. HH and JW performed experiments and analyzed the data. RS and JJLW assisted with bioinformatics. HH, VA, and JW wrote the manuscript. All authors reviewed the results and approved the final version of the manuscript.

## FUNDING INFORMATION

This work was supported by grants from the Australian Medical Research Future Fund (Clem Jones Centre for Ageing Dementia Research Flagship Project Grant) to VA, and a University of Queensland (UQ) Early Career Researcher Grant and UQ Amplify Fellowship to JW. HH is a recipient of a UQ Research Training Scholarship.

## CONFLICT OF INTEREST

None declared.

## Supporting information


**Appendix S1.** Extended Methods

**Table S1.** List of differentially methylated peaks

Table S2. KEGG pathways associated with differentially methylated genes

Figure S1. Validation of aging‐related gene expression changes in the hippocampus

Figure S2. m6A peaks in young and aged animals

## Data Availability

The sequenced data from the RNA‐Seq and MeRIP‐Seq experiments are available in the NCBI GEO repository under the accession code GSE217884.
