# Supplementary material for: The N6‐methyladenosine RNA landscape in the aged mouse hippocampus
Source: Aging Cell. 2022 Dec 9;22(1):e13755. doi: 10.1111/acel.13755 (PMC9835576; doi:10.1111/acel.13755)
Supplement: Supplementary file 1 — Appendix S1. Extended Methods [file ACEL-22-e13755-s002.pdf]

Extended Methods

MeRIP-seq and RNA-seq analyses

Quality control of sequencing reads. Sequenced reads (22-49 million total coverages) were subjected to Trimmomatic (version 0.36) (Bolger, Lohse, & Usadel, 2014) to trim off low quality reads and remove adaptors. All reads were then aligned against a custom contaminant list to further filter reads mapped to rRNA or Phi X genomes using Sortmerna (version 2.1) (Kopylova, Noe, & Touzet, 2012) or Bowtie2 (version 2.2.7) (Langmead & Salzberg, 2012).

Alignment and reads coverage. Sequenced reads were aligned to the mouse genome (mm10) with STAR (version 2.5.3a) (Dobin et al., 2013) using reference annotation GRCm38.86 or the later GRCm39 (for RNA-seq). Only uniquely mapped reads were selected by Samtools (version 1.3) to minimize the rate of false positives. Deeptools (Ramirez et al., 2016) was used to generate a bigwig format to facilitate visualization of the reads coverage and comparison among different samples.

m6A site detection and characterization. Macs2 (version 2.2.1) (Zhang et al., 2008) was used to detect m6A sites by setting the effective genome size as 1.87×10^9, and *shiftsize* based on the length of RNA fragments (option of *—nomodel*). Peaks with fold enrichment over 3 were selected and recentered (+-100bp of summit). All the filtered peaks were chosen for de novo motif analysis with MEME-CHIP (Machanick & Bailey, 2011). Peak counts were further generated by Subread (1.6.4) (Liao, Smyth, & Shi, 2014) and annotated with Bedtools (version 2.25.0) (Quinlan & Hall, 2010). Peaks were assigned to 6 non-overlapping regions: coding sequence (CDS), 5'UTR, 3'UTR, intron, start codon, and stop codon.

Differential m6A and gene expression analysis. Differentially expressed genes and differential changes in m6A peaks between young and aged samples were analyzed using the EdgeR likelihood ratio test (Robinson, McCarthy, & Smyth, 2010) (FC > 1.5; FDR < 0.05) with the additional option to remove unwanted sources of variation with RUV (Risso, Ngai, Speed, & Dudoit, 2014). GO enrichment analysis based on biological processes and Kyoto Encyclopedia of Genes and Genomes (KEGG) were performed using the 'clusterProfiler' R package (Wu et al., 2021).

Generation of Gpr17 reporter constructs

Gene blocks (~400 nucleotide long) containing wildtype or mutated sequence of mouse *Gpr17* 3'UTR around Peak 1 or Peak 2 were ordered from Integrated DNA Technologies as detailed below (underlined adenine sites were mutated to thymine in the mutant constructs).

3-UTR-Peak1:
GCATGCGCTAGCTGGCGTTAATTAAATCCAGTCTCAGCGAATCCATCAAGAGGCAGG**A**CTA**A**CC
ACAGGGATGCCCTGCCCACCCCTCCACAGGACTGGGTTGGCCTGGCTTCTGTACAGCTCCCAG
ACACTCAGTG**A**CTTCACTCGTGCTAAATAGGGAAGAGAGCCACAGGG**A**CATTTCTGGA**A**CAATG
GGAATCTTTCTTCTCTAATAAATTTCTAGCTTCTTTCATACTACAGATGCCTAGGAGTTGAGGAATT
CCCCCCGGGATCCCTAGGCCCACATGAGGATCACCCATGTGTGCAGGGCCTAGCAAGTTAAAAT
AAGGCTAGTCCGTTATCAACTTGGCCAACATGAGGATCACCCATGTCTGCGTGGCCCGCTCGAG
GCTCTAGAATGC

3-UTR-Peak2:
GCATGCGCTAGCTGGCGTTAATTAAATCCAGTCTCCAAG**A**CTGTGCCAGCCAGG**A**CCAGCACGG
G**A**CCATGTGTCTCTTCGACAGAAGAGCAAAGGG**A**CAAGAGTACCGCAGTCTGTG**A**CAGGAGGG

ACAGAGGGGGCAAAGACCCAGACAGGAAGGACAGTCTCCTGGGAAACTGGAGGGTCTCTGGA
AATTAGCACTGCCTTTCCTGACACAGGGGTTCAGAAGCAGACTTGGGTCAGAGGAGATGCCTAG
GAGTTGAGGAATTCCCCCCGGGATCCCTAGGCCCACATGAGGATCACCCATGTGTGCAGGGCC
TAGCAAGTTAAAATAAGGCTAGTCCGTTATCAACTTGGCCAACATGAGGATCACCCATGTCTGCG
TGGCCCGCTCGAGGCTCTAGAATGC

The amplified gene block sequence was cloned into pIS0 (Yekta, Shih, & Bartel, 2004) downstream *Fluc* open reading frame using the *Nhe*I and *Xba*I restriction sites to generate Fluc-Gpr17(3'UTR):Peak1 or Peak2 wild-type or mutant.

The tetracycline-inducible firefly luciferase constructs were generated by subcloning the *Fluc*-3'UTR-Gpr17 from the corresponding pISO luciferase plasmids into the *BamH*I and *Xba*I sites in pBI-Tet vector (Clontech) after amplification using

a forward primer: GGCTTTTGCAAAAGGATCCGCATTCCGGTACTGTTGG; and

a reverse primer: GGGCCTAGGGATTCTAGAGAATTCCTCAACTCCTAGGCATCT.

Oligonucleotides

List of primers used in the qPCR analyses.

Name	Sequence (5' to 3')
Gpr17F	GTCTCTGGAAGTCAGCACGC
Gpr17R	AGACAAGTTCTCGCATCAGGC
Gpr17peak1F	ATCAAGAGGCAGGACTAACCAC
Gpr17peak1R	AAATGTCCCTGTGGCTCTCTTC
Gpr17peak2F	CAAGAGTACCGCAGTCTGTGA
Gpr17peak2R	CTCTGACCCAAGTCTGCTTCTG
Neat1F	CATGTGTTATGGTGGGGCCT
Neat1R	GCAGAGGGAATAGGTACGGC
C4bF	GGGAAACCAGTGACAACAAGG
C4bR	CCTGTAGAGCAGAGCCTCTAA
Cldn2F	CAACTGGTGGGCTACATCCTA
Cldn2R	CCCTTGGAAAAGCCAACCG
Trem2F	CTGGAACCGTCACCATCACTC
Trem2R	CGAAACTCGATGACTCCTCGG
Rpl13aF	TACGCTGTGAAGGCATCAAC
Rpl13aR	GGGAGGGGTTGGTATTCATC
FlucF	ACTGGGACGAAGACGAACAC
FlucR	GGGTGTTGGAGCAAGATGGA
ActbF	CTTCGCGGGCGACGAT
ActbR	CCACATAGGAATCCTTCTGACC

References

Bolger, A. M., Lohse, M., & Usadel, B. (2014). Trimmomatic: a flexible trimmer for Illumina sequence data. *Bioinformatics, 30*(15), 2114-2120. doi:10.1093/bioinformatics/btu170

Dobin, A., Davis, C. A., Schlesinger, F., Drenkow, J., Zaleski, C., Jha, S., . . . Gingeras, T. R. (2013). STAR: ultrafast universal RNA-seq aligner. *Bioinformatics, 29*(1), 15-21. doi:10.1093/bioinformatics/bts635

Kopylova, E., Noe, L., & Touzet, H. (2012). SortMeRNA: fast and accurate filtering of ribosomal RNAs in metatranscriptomic data. *Bioinformatics, 28*(24), 3211-3217. doi:10.1093/bioinformatics/bts611

Langmead, B., & Salzberg, S. L. (2012). Fast gapped-read alignment with Bowtie 2. *Nat Methods, 9*(4), 357-359. doi:10.1038/nmeth.1923

Liao, Y., Smyth, G. K., & Shi, W. (2014). featureCounts: an efficient general purpose program for assigning sequence reads to genomic features. *Bioinformatics (Oxford, England), 30*(7), 923-930. doi:10.1093/bioinformatics/btt656

Machanick, P., & Bailey, T. L. (2011). MEME-ChIP: motif analysis of large DNA datasets. *Bioinformatics (Oxford, England), 27*(12), 1696-1697. doi:10.1093/bioinformatics/btr189

Quinlan, A. R., & Hall, I. M. (2010). BEDTools: a flexible suite of utilities for comparing genomic features. *Bioinformatics (Oxford, England), 26*(6), 841-842. doi:10.1093/bioinformatics/btq033

Ramirez, F., Ryan, D. P., Gruning, B., Bhardwaj, V., Kilpert, F., Richter, A. S., . . . Manke, T. (2016). deepTools2: a next generation web server for deep-sequencing data analysis. *Nucleic Acids Research, 44*(W1), W160-165. doi:10.1093/nar/gkw257

Risso, D., Ngai, J., Speed, T. P., & Dudoit, S. (2014). Normalization of RNA-seq data using factor analysis of control genes or samples. *Nat Biotechnol, 32*(9), 896-902. doi:10.1038/nbt.2931

Robinson, M. D., McCarthy, D. J., & Smyth, G. K. (2010). edgeR: a Bioconductor package for differential expression analysis of digital gene expression data. *Bioinformatics, 26*(1), 139-140. doi:10.1093/bioinformatics/btp616

Wu, T., Hu, E., Xu, S., Chen, M., Guo, P., Dai, Z., . . . Yu, G. (2021). clusterProfiler 4.0: A universal enrichment tool for interpreting omics data. *Innovation (Camb), 2*(3), 100141. doi:10.1016/j.xinn.2021.100141

Yekta, S., Shih, I. H., & Bartel, D. P. (2004). MicroRNA-directed cleavage of HOXB8 mRNA. *Science, 304*(5670), 594-596. doi:10.1126/science.1097434

Zhang, Y., Liu, T., Meyer, C. A., Eeckhoute, J., Johnson, D. S., Bernstein, B. E., . . . Liu, X. S. (2008). Model-based analysis of ChIP-Seq (MACS). *Genome Biol, 9*(9), R137. doi:10.1186/gb-2008-9-9-r137
