# Supplementary material for: The N6‐methyladenosine RNA landscape in the aged mouse hippocampus
Source: Aging Cell. 2022 Dec 9;22(1):e13755. doi: 10.1111/acel.13755 (PMC9835576; doi:10.1111/acel.13755)
Supplement: Supplementary file 2 — Table S1. List of differentially methylated peaks [file ACEL-22-e13755-s005.pdf]

Chr	Start	End	IP_Y1	IP_Y2	IP_Y3	IP_A1	IP_A2	IP_A3	m6alogFC	m6alogCPM	m6aLR	m6aPValue	m6aFDR	strand	type	genename	function
19	6531447	6531663	39	26	115	15	9	5	-1.66209186	1.561681186	24.39957991	7.83E-07	5.69E-05	+	Exon	Nrxn2	protein_coding
X	170673335	170673535	108	114	744	82	23	65	-1.469979049	3.835525366	13.57124389	0.000229677	0.003968756	+	Intron	Asmt	protein_coding
3	132839354	132839554	97	70	267	37	28	31	-1.244433239	2.834527453	22.81846474	1.78E-06	0.000106013	+	3'UTR	Tbck	protein_coding
X	139809371	139809571	432	91	726	66	23	162	-1.202532883	4.248706755	10.90595694	0.000958555	0.011255644	+	Exon	Cldn2	protein_coding
X	139809371	139809571	432	91	726	66	23	162	-1.202532883	4.248706755	10.90595694	0.000958555	0.011255644	+	Stop codon	Cldn2	protein_coding
X	139809371	139809571	432	91	726	66	23	162	-1.202532883	4.248706755	10.90595694	0.000958555	0.011255644	+	3'UTR	Cldn2	protein_coding
7	29965372	29965572	112	67	321	65	28	30	-1.061294888	3.046092128	16.57175862	4.68E-05	0.001221637	+	Exon	C230062I16Rik	processed_transcript
7	29965372	29965572	112	67	321	65	28	30	-1.061294888	3.046092128	16.57175862	4.68E-05	0.001221637	-	Exon	Gm10169	processed_pseudogene
10	3957576	3957776	104	60	160	37	21	33	-1.018662698	2.518110248	21.28753465	3.95E-06	0.000197327	+	Exon	Plekhg1	protein_coding
18	31945466	31945668	2726	1806	3878	1194	570	743	-1.003718247	7.133056417	50.68360957	1.09E-12	7.66E-10	-	3'UTR	Gpr17	protein_coding
5	53653565	53653768	73	42	109	29	12	24	-0.988208183	2.023501159	16.12037239	5.94E-05	0.001471488	+	Exon	Rbpj	protein_coding
5	53653565	53653768	73	42	109	29	12	24	-0.988208183	2.023501159	16.12037239	5.94E-05	0.001471488	+	Stop codon	Rbpj	protein_coding
5	53653565	53653768	73	42	109	29	12	24	-0.988208183	2.023501159	16.12037239	5.94E-05	0.001471488	+	3'UTR	Rbpj	protein_coding
X	134973262	134973462	188	207	322	141	50	58	-0.882615235	3.700685743	15.70911118	7.39E-05	0.001690061	-	Exon	Zmat1	protein_coding
18	31946588	31946788	2681	1618	3224	1114	508	854	-0.873679656	7.021952396	42.45897732	7.22E-11	2.64E-08	-	3'UTR	Gpr17	protein_coding
5	124340537	124340737	379	328	484	175	102	123	-0.867272031	4.421553956	29.08215283	6.94E-08	8.26E-06	+	Exon	2810006K23Rik	protein_coding
1	162648698	162648921	998	929	838	356	239	349	-0.83799753	5.650499539	23.56860497	1.21E-06	8.08E-05	+	Exon	Myoc	protein_coding
1	162648698	162648921	998	929	838	356	239	349	-0.83799753	5.650499539	23.56860497	1.21E-06	8.08E-05	-	Intron	7420461P10Rik	protein_coding
18	31946133	31946335	743	552	984	340	171	264	-0.835410574	5.337816466	33.4472359	7.32E-09	1.41E-06	-	3'UTR	Gpr17	protein_coding
5	110128791	110128991	179	102	287	82	53	45	-0.825417746	3.34098219	16.92580116	3.89E-05	0.001085276	+	Exon	Zfp605	protein_coding
11	77454686	77454886	179	110	193	65	49	48	-0.82120125	3.152949714	17.41747868	3.00E-05	0.000895556	+	Exon	Ssh2	protein_coding
3	35894941	35895141	571	263	526	222	83	165	-0.810862933	4.597918453	19.36523015	1.08E-05	0.000418441	-	3'UTR	Dcun1d1	protein_coding
18	37686571	37686771	333	286	566	139	97	162	-0.800216958	4.414940212	15.71695691	7.36E-05	0.00168697	+	Exon	Pcdhga4	protein_coding
X	94637096	94637328	552	376	652	289	83	204	-0.797119617	4.830652192	17.70450179	2.58E-05	0.000809595	+	Exon	Gspt2	protein_coding
13	14186896	14187138	1467	827	1643	580	320	463	-0.773759517	6.128358201	31.35752379	2.15E-08	3.17E-06	+	Exon	Arid4b	protein_coding
4	40976662	40976862	381	410	442	207	108	140	-0.767513336	4.52087909	24.42141466	7.74E-07	5.67E-05	+	Exon	Nfx1	protein_coding
7	29776101	29776301	236	120	276	111	47	62	-0.766462998	3.52287908	17.54321352	2.81E-05	0.000859895	+	Exon	Zfp84	protein_coding
X	13156175	13156382	2895	1589	3654	1190	627	1024	-0.740428943	7.168210886	30.82364395	2.83E-08	3.91E-06	+	Exon	Usp9x	protein_coding
17	32305632	32305832	190	275	195	150	50	58	-0.73995545	3.658069178	14.23279235	0.000161531	0.003058568	-	Exon	Akap8	protein_coding
7	12810082	12810282	265	156	307	96	51	112	-0.736761607	3.746312245	14.87359273	0.000114961	0.002362319	-	Exon	Zfp329	protein_coding
2	140661277	140661477	407	184	366	115	73	147	-0.732577291	4.129071416	11.86249831	0.000572757	0.007723228	-	Exon	Flrt3	protein_coding
17	94816303	94816503	768	632	988	340	216	310	-0.73061078	5.452189116	21.82925681	2.98E-06	0.000160089	-	Intron	Gm1976	lincRNA
1	179753845	179754045	379	230	380	150	89	123	-0.717714202	4.197182369	18.71638748	1.52E-05	0.000537504	-	Exon	Ahctf1	protein_coding
1	179753845	179754045	379	230	380	150	89	123	-0.717714202	4.197182369	18.71638748	1.52E-05	0.000537504	-	3'UTR	Ahctf1	protein_coding
11	111024352	111024552	240	112	476	120	69	74	-0.707871396	3.856426357	13.92891712	0.000189855	0.00344317	+	Exon	Kcnj16	protein_coding
11	111024352	111024552	240	112	476	120	69	74	-0.707871396	3.856426357	13.92891712	0.000189855	0.00344317	+	Start codon	Kcnj16	protein_coding
11	111024352	111024552	240	112	476	120	69	74	-0.707871396	3.856426357	13.92891712	0.000189855	0.00344317	+	5'UTR	Kcnj16	protein_coding
5	104522115	104522315	284	132	306	149	59	57	-0.705266614	3.727887079	10.86293451	0.000981081	0.011462182	+	Exon	BC005561	protein_coding
9	20593827	20594027	245	170	345	136	48	100	-0.704963573	3.813983785	15.515154	8.18E-05	0.001816356	+	Exon	Zfp846	protein_coding
9	20593827	20594027	245	170	345	136	48	100	-0.704963573	3.813983785	15.515154	8.18E-05	0.001816356	+	3'UTR	Zfp846	protein_coding
14	45280015	45280215	261	135	298	116	47	90	-0.704708643	3.677353715	15.91773199	6.62E-05	0.001587322	+	Exon	Gpr137c	protein_coding
14	45280015	45280215	261	135	298	116	47	90	-0.704708643	3.677353715	15.91773199	6.62E-05	0.001587322	+	Stop codon	Gpr137c	protein_coding
14	45280015	45280215	261	135	298	116	47	90	-0.704708643	3.677353715	15.91773199	6.62E-05	0.001587322	+	3'UTR	Gpr137c	protein_coding
3	95327484	95327684	442	339	472	227	108	145	-0.702931464	4.549521641	21.40790189	3.71E-06	0.00018626	-	Exon	Setdb1	protein_coding
9	58199704	58199904	578	572	669	314	152	237	-0.701203192	5.092200862	22.93105105	1.68E-06	0.000101201	-	Exon	Islr2	protein_coding
5	104521856	104522056	306	248	276	139	83	95	-0.701187064	3.979864515	17.00328084	3.73E-05	0.001069056	+	Exon	BC005561	protein_coding
16	36912229	36912429	434	336	443	215	130	114	-0.700804752	4.508178407	15.51505646	8.19E-05	0.001816356	+	Exon	Golgb1	protein_coding
10	90601841	90602070	158	129	306	79	51	81	-0.695998265	3.458586664	11.08704618	0.000869328	0.010441225	+	Intron	Anks1b	protein_coding
19	44941409	44941609	200	128	305	86	69	67	-0.694479084	3.549469516	12.7915592	0.000348187	0.00534391	+	Exon	Fam178a	protein_coding
1	66414618	66414818	4492	3469	5914	2465	1181	1630	-0.691609202	7.98305205	27.75279474	1.38E-07	1.47E-05	+	Exon	Map2	protein_coding
18	34314611	34314849	1156	515	1195	428	236	363	-0.68885882	5.687994081	19.8241761	8.49E-06	0.000352593	+	Exon	Apc	protein_coding
10	95412755	95412955	425	249	512	222	93	131	-0.684446204	4.44619131	18.20912016	1.98E-05	0.000674596	-	Exon	Socs2	protein_coding
X	108743404	108743608	513	291	753	227	125	203	-0.682178	4.813486308	19.70709916	9.03E-06	0.000367763	-	Exon	Brwd3	protein_coding
X	108743404	108743608	513	291	753	227	125	203	-0.682178	4.813486308	19.70709916	9.03E-06	0.000367763	-	3'UTR	Brwd3	protein_coding
10	43582895	43583095	744	437	888	327	137	313	-0.681946556	5.24052182	15.67799205	7.51E-05	0.00171018	+	3'UTR	Cd24a	protein_coding
17	17387158	17387358	645	554	809	392	149	253	-0.68128532	5.225900318	19.38412031	1.07E-05	0.000415954	+	Exon	Chd1	protein_coding
14	88468117	88468404	1532	1139	1858	789	395	548	-0.680612553	6.381156002	26.83423348	2.22E-07	2.15E-05	-	Exon	Pcdh20	protein_coding
15	12374672	12374872	295	226	407	141	63	148	-0.680609522	4.115680692	13.07288939	0.000299601	0.004760649	-	Exon	Pdzd2	protein_coding
5	49961149	49961349	267	193	288	110	59	116	-0.680490553	3.823618263	14.07029934	0.000176103	0.003253457	-	Exon	Adgra3	protein_coding
2	104147606	104147806	261	164	267	148	49	75	-0.680133634	3.705766126	13.09361001	0.000296305	0.004731303	-	3'UTR	D430041D05Rik	protein_coding
3	108151662	108151862	325	205	450	129	108	111	-0.678787369	4.176263999	13.33237674	0.000260863	0.00431165	-	5'UTR	Gpr61	protein_coding
17	87437692	87437892	184	332	133	110	46	88	-0.674075409	3.644320027	13.85675937	0.000197286	0.00353658	-	Intron	Calm2	protein_coding
3	108618961	108619161	620	379	603	302	129	192	-0.673579266	4.897242617	18.33542929	1.85E-05	0.000637915	+	Exon	Wdr47	protein_coding
11	100463177	100463377	330	297	375	160	115	107	-0.673506811	4.251153643	14.26812881	0.000158526	0.003013223	-	Exon	Klhl11	protein_coding
18	69985210	69985410	313	181	351	110	99	95	-0.670562647	3.978635666	11.60698614	0.000657045	0.008556307	-	Exon	Rab27b	protein_coding
3	103353711	103353911	230	198	304	144	83	56	-0.668488992	3.796766198	9.452305014	0.002108839	0.02002283	+	Exon	Trim33	protein_coding

3	103353711	103353911	230	198	304	144	83	56	-0.668488992	3.796766198	9.452305014	0.002108839	0.02002283	+	Stop codon	Trim33	protein_coding
3	103353711	103353911	230	198	304	144	83	56	-0.668488992	3.796766198	9.452305014	0.002108839	0.02002283	+	3'UTR	Trim33	protein_coding
15	88730253	88730453	542	506	889	314	195	221	-0.668353969	5.16786618	14.30250129	0.000155658	0.002988101	-	Exon	Brd1	protein_coding
15	16855736	16855940	409	275	432	224	105	107	-0.667193881	4.388908323	14.39075057	0.00014853	0.002889904	+	Exon	Cdh9	protein_coding
19	47733428	47733631	1470	1270	1944	857	415	560	-0.664760176	6.441626045	22.78297858	1.81E-06	0.000107342	+	Exon	Sfr1	protein_coding
3	95765082	95765349	519	394	641	258	115	231	-0.664074615	4.859102204	16.79440984	4.17E-05	0.001137365	-	Exon	Rprd2	protein_coding
10	85114613	85114813	129	101	142	73	45	27	-0.662887941	2.855297086	8.83454963	0.002955806	0.025207234	+	Exon	Tmem263	protein_coding
10	85114613	85114813	129	101	142	73	45	27	-0.662887941	2.855297086	8.83454963	0.002955806	0.025207234	+	Stop codon	Tmem263	protein_coding
10	85114613	85114813	129	101	142	73	45	27	-0.662887941	2.855297086	8.83454963	0.002955806	0.025207234	+	3'UTR	Tmem263	protein_coding
19	47619954	47620250	779	371	803	315	143	274	-0.660506125	5.157696194	16.11535743	5.96E-05	0.001471488	+	Exon	Slk	protein_coding
14	88468804	88469259	3578	2365	3934	1751	826	1269	-0.656707349	7.507143245	26.06234057	3.31E-07	2.88E-05	-	Exon	Pcdh20	protein_coding
5	90253724	90253926	1182	662	1300	515	227	461	-0.656452493	5.849313418	16.92827649	3.88E-05	0.001085276	-	Exon	Ankrd17	protein_coding
1	143640808	143641008	247	140	242	99	73	64	-0.656076809	3.576389536	11.15016594	0.000840242	0.01019012	+	5'UTR	B3galt2	protein_coding
5	49960565	49960766	784	431	840	359	153	278	-0.654938464	5.242174501	18.06664925	2.13E-05	0.000722023	-	Exon	Adgra3	protein_coding
6	137380325	137380525	562	431	778	297	149	237	-0.652559883	5.041818883	18.79773296	1.45E-05	0.000524078	+	Exon	Ptpro	protein_coding
4	147826420	147826620	397	208	466	158	73	171	-0.647696749	4.308476897	12.37759508	0.000434516	0.006315647	-	Exon	Zfp933	protein_coding
2	106969183	106969383	1345	772	1772	617	358	464	-0.64675496	6.143518759	22.36640156	2.25E-06	0.00012871	-	Exon	Arl14ep	protein_coding
3	18056128	18056349	2256	1714	3259	1132	613	1019	-0.645903529	7.054015706	18.87155066	1.40E-05	0.000506229	+	3'UTR	Bhlhe22	protein_coding
9	107852981	107853304	1100	696	1303	428	311	414	-0.644101613	5.83972003	15.76618023	7.17E-05	0.001671459	-	Exon	Rbm6	protein_coding
9	107852981	107853304	1100	696	1303	428	311	414	-0.644101613	5.83972003	15.76618023	7.17E-05	0.001671459	-	Start codon	Rbm6	protein_coding
9	107852981	107853304	1100	696	1303	428	311	414	-0.644101613	5.83972003	15.76618023	7.17E-05	0.001671459	-	5'UTR	Rbm6	protein_coding
8	86861731	86861931	781	441	1021	359	187	292	-0.643997822	5.356736663	20.91146757	4.81E-06	0.00023536	-	Exon	N4bp1	protein_coding
9	80113803	80114003	1015	567	1048	390	257	341	-0.643043105	5.604712202	16.66281826	4.46E-05	0.001195926	+	Exon	Senp6	protein_coding
9	80113803	80114003	1015	567	1048	390	257	341	-0.643043105	5.604712202	16.66281826	4.46E-05	0.001195926	+	3'UTR	Senp6	protein_coding
17	33003252	33003452	309	186	349	140	75	109	-0.641844392	3.98966911	15.51252905	8.20E-05	0.001816356	+	Exon	Zfp952	protein_coding
2	37352300	37352507	993	1023	1304	581	313	430	-0.638394589	5.971729436	18.89600517	1.38E-05	0.000503469	-	Exon	Pdcl	protein_coding
2	37352300	37352507	993	1023	1304	581	313	430	-0.638394589	5.971729436	18.89600517	1.38E-05	0.000503469	-	3'UTR	Pdcl	protein_coding
18	32079735	32079966	258	195	256	106	61	113	-0.635401106	3.771144341	11.91956507	0.000555474	0.007510684	+	Exon	Iws1	protein_coding
2	82257486	82257686	364	291	358	194	96	119	-0.635090316	4.27943954	16.02348787	6.26E-05	0.001513296	+	Exon	Zfp804a	protein_coding
4	94965372	94965572	486	246	578	224	111	158	-0.634530293	4.592262264	17.29392577	3.20E-05	0.000938373	-	Exon	Mysm1	protein_coding
5	14712554	14712754	1610	1559	2096	1009	502	614	-0.63155513	6.632031355	18.95537753	1.34E-05	0.000491671	+	Exon	Pclo	protein_coding
12	41454026	41454238	1861	1169	2409	894	494	687	-0.630549293	6.64158922	23.07510894	1.56E-06	9.68E-05	-	Exon	Lrrn3	protein_coding
14	20695943	20696143	259	165	380	153	40	124	-0.629025722	3.909785965	8.789340529	0.003029958	0.025640498	+	Exon	Fut11	protein_coding
11	29503925	29504125	563	363	553	260	139	188	-0.623366287	4.806453434	16.64569084	4.51E-05	0.001200268	+	Exon	Ccdc88a	protein_coding
18	31945017	31945232	402	602	439	315	138	147	-0.621973576	4.807851161	13.3956545	0.000252208	0.004225124	-	3'UTR	Gpr17	protein_coding
19	5801870	5802078	17140	8335	39474	11364	4559	6235	-0.619490166	10.10752383	10.40567241	0.001256288	0.01370061	-	Exon	Malat1	lincRNA
19	5801870	5802078	17140	8335	39474	11364	4559	6235	-0.619490166	10.10752383	10.40567241	0.001256288	0.01370061	+	Exon	Gm37376	TEC
8	110975817	110976017	168	72	179	74	29	56	-0.61791782	2.989484977	9.446534199	0.002115485	0.020066651	-	3'UTR	Ddx19a	protein_coding
4	62689672	62689872	202	116	213	87	38	84	-0.617239398	3.347077733	10.45709632	0.001221792	0.013403097	+	Exon	Rgs3	protein_coding
10	87491690	87491890	383	344	579	251	121	153	-0.615831383	4.628647387	13.55172856	0.000232078	0.00399826	-	3'UTR	Ascl1	protein_coding
8	123126193	123126413	693	1577	745	638	286	284	-0.614138011	5.858818633	11.82960455	0.000582964	0.007807606	+	Exon	Cpne7	protein_coding
13	38193247	38193447	233	371	177	150	49	119	-0.612047733	3.932361372	10.58102873	0.001142541	0.012717198	+	Exon	Dsp	protein_coding
2	11767559	11767759	174	164	257	87	68	78	-0.61082997	3.525292895	9.596484164	0.001949503	0.018909604	-	Exon	Fbxo18	protein_coding
12	100130952	100131152	548	455	608	283	185	175	-0.609250319	4.944768065	13.08061947	0.000298367	0.004748884	-	Exon	Nrde2	protein_coding
13	38192969	38193169	143	261	155	137	44	55	-0.609017599	3.467024126	9.18629469	0.00243834	0.022130902	+	Exon	Dsp	protein_coding
X	103185712	103185925	11356	7493	15462	5406	3069	4768	-0.60891507	9.301515388	20.22437216	6.89E-06	0.00030282	-	Exon	Nap1l2	protein_coding
12	105836987	105837187	383	314	447	258	97	125	-0.605028557	4.454621559	12.35655326	0.000439441	0.006359352	+	3'UTR	Papola	protein_coding
X	94636542	94636766	436	688	610	439	138	184	-0.603816516	5.076454671	8.3496323	0.003857654	0.030357525	+	Exon	Gspt2	protein_coding
5	106636627	106636847	1221	542	1290	562	218	414	-0.602204012	5.806025575	13.38858292	0.000253161	0.004233907	-	Exon	Zfp644	protein_coding
6	136560704	136560904	855	514	978	387	236	292	-0.599905514	5.456498593	16.80317584	4.15E-05	0.001137365	+	Exon	Atf7ip	protein_coding
7	27748211	27748411	404	201	475	179	91	146	-0.594538315	4.332756154	14.14252387	0.000169469	0.003172412	+	Exon	Zfp60	protein_coding
7	27748211	27748411	404	201	475	179	91	146	-0.594538315	4.332756154	14.14252387	0.000169469	0.003172412	+	3'UTR	Zfp60	protein_coding
3	152427617	152427817	754	450	906	361	171	305	-0.59384111	5.301024294	16.27575369	5.48E-05	0.001404689	+	Exon	Zzz3	protein_coding
2	69795747	69795947	337	190	324	173	70	105	-0.593038609	4.02314448	11.56903663	0.000670592	0.008652907	+	Exon	Phospho2	protein_coding
5	107503469	107503716	1830	1073	1811	850	450	591	-0.592842439	6.466109526	17.31617082	3.16E-05	0.000932035	+	Exon	A830010M20Rik	protein_coding
X	36611933	36612133	300	197	419	182	82	101	-0.591676731	4.111881203	12.17859059	0.000483412	0.006777445	-	Exon	Akap17b	protein_coding
7	79559274	79559474	217	98	254	106	50	62	-0.591594396	3.421696169	10.25415122	0.001363774	0.014478559	+	Exon	Gm35040	lincRNA
9	59315366	59315566	295	197	276	145	57	117	-0.591476749	3.893481922	11.04939495	0.000887161	0.010641631	+	3'UTR	Adpgk	protein_coding
10	69992964	69993164	455	239	540	252	97	143	-0.590388328	4.525768739	12.59266737	0.000387263	0.005834921	+	Exon	Ank3	protein_coding
6	65704205	65704440	690	300	764	271	157	235	-0.587940151	5.015492253	14.12207664	0.000171321	0.003182969	+	Exon	Ndnf	protein_coding
7	7115267	7115467	240	147	242	120	73	60	-0.587247355	3.603643251	9.041135404	0.002639716	0.023526562	-	Exon	Zfp954	protein_coding
7	7115267	7115467	240	147	242	120	73	60	-0.587247355	3.603643251	9.041135404	0.002639716	0.023526562	-	Stop codon	Zfp954	protein_coding
7	7115267	7115467	240	147	242	120	73	60	-0.587247355	3.603643251	9.041135404	0.002639716	0.023526562	-	3'UTR	Zfp954	protein_coding
14	75962492	75962723	703	572	869	337	179	356	-0.586460276	5.352629506	11.60207652	0.000658782	0.008556378	+	Exon	Kctd4	protein_coding
14	75962492	75962723	703	572	869	337	179	356	-0.586460276	5.352629506	11.60207652	0.000658782	0.008556378	+	Start codon	Kctd4	protein_coding
14	75962492	75962723	703	572	869	337	179	356	-0.586460276	5.352629506	11.60207652	0.000658782	0.008556378	+	5'UTR	Kctd4	protein_coding
18	77152503	77152723	325	320	449	254	100	113	-0.584282974	4.402497753	9.677501733	0.001865381	0.018309261	+	Exon	Pias2	protein_coding

15	71463576	71463776	412	241	457	216	82	155	-0.584176177	4.394562745	12.64428619	0.000376715	0.005693354	-	Exon	Fam135b	protein_coding
18	36644184	36644384	439	327	548	211	157	150	-0.583625961	4.648204164	10.86829515	0.000978245	0.011443653	+	Exon	Ankhd1	protein_coding
3	116924087	116924287	512	385	582	234	134	232	-0.582834123	4.8240782	12.85826289	0.000335992	0.005188982	-	Exon	Palmd	protein_coding
12	41453301	41453501	1268	823	1299	569	353	437	-0.582172048	6.00444233	15.52679127	8.13E-05	0.001816356	-	Exon	Lrrn3	protein_coding
9	51849186	51849386	505	334	624	304	136	158	-0.581715064	4.790526486	12.69424752	0.000366782	0.005551723	+	Exon	Arhgap20	protein_coding
13	58004171	58004371	336	203	513	217	91	107	-0.580523094	4.293998934	10.32295453	0.001313858	0.014141351	-	3'UTR	Klhl3	protein_coding
11	77983058	77983270	747	982	567	1013	447	696	0.580260222	6.038930204	17.2006956	3.36E-05	0.000971965	+	5'UTR	Phf12	protein_coding
6	39871824	39872039	498	594	509	866	302	411	0.580538715	5.527366391	12.18280867	0.00048232	0.006777445	-	Exon	Gm26833	antisense
8	82863739	82863940	210	211	211	292	153	152	0.580880898	4.17787613	11.77923673	0.00059895	0.007957024	+	5'UTR	Rnf150	protein_coding
5	112217446	112217647	590	1143	376	823	478	500	0.580892316	5.872100686	13.84133764	0.000198912	0.003539866	-	Exon	Gm27627	misc_RNA
5	112217446	112217647	590	1143	376	823	478	500	0.580892316	5.872100686	13.84133764	0.000198912	0.003539866	-	Exon	Miat	lincRNA
2	84711414	84711614	497	558	453	576	291	543	0.581615715	5.435490758	12.37837599	0.000434335	0.006315647	-	5'UTR	Zdhhc5	protein_coding
10	127079529	127079729	6680	10253	5608	8948	4084	7752	0.582111742	9.32957529	13.37560714	0.000254918	0.004248921	+	Exon	Agap2	protein_coding
10	127079529	127079729	6680	10253	5608	8948	4084	7752	0.582111742	9.32957529	13.37560714	0.000254918	0.004248921	+	Start codon	Agap2	protein_coding
10	127079529	127079729	6680	10253	5608	8948	4084	7752	0.582111742	9.32957529	13.37560714	0.000254918	0.004248921	+	5'UTR	Agap2	protein_coding
7	19394911	19395227	296	385	430	449	226	368	0.582348757	4.9896979	11.49487685	0.000697883	0.008906591	+	3'UTR	Ercc2	protein_coding
7	19394911	19395227	296	385	430	449	226	368	0.582348757	4.9896979	11.49487685	0.000697883	0.008906591	-	Exon	Klc3	protein_coding
9	43222541	43222741	238	171	260	280	116	228	0.582402069	4.233265216	12.50349897	0.000406191	0.006037274	-	Exon	Oaf	protein_coding
9	43222541	43222741	238	171	260	280	116	228	0.582402069	4.233265216	12.50349897	0.000406191	0.006037274	-	Stop codon	Oaf	protein_coding
9	43222541	43222741	238	171	260	280	116	228	0.582402069	4.233265216	12.50349897	0.000406191	0.006037274	-	3'UTR	Oaf	protein_coding
16	35155064	35155288	838	1011	940	1082	609	901	0.582592589	6.324012713	15.72475515	7.33E-05	0.00168697	+	5'UTR	Adcy5	protein_coding
7	25724758	25724958	402	657	328	666	272	363	0.583141308	5.304932378	16.60562887	4.60E-05	0.001209587	-	Exon	Hnrnpul1	protein_coding
10	105573894	105574106	226	237	177	320	124	176	0.583159472	4.202914051	12.45164132	0.000417625	0.006133436	-	5'UTR	Tmtc2	protein_coding
10	105573894	105574106	226	237	177	320	124	176	0.583159472	4.202914051	12.45164132	0.000417625	0.006133436	+	Exon	Gm15663	processed_transcript
10	19934469	19934679	184	216	222	265	130	193	0.58339736	4.155444293	13.40535095	0.000250908	0.004211658	+	Exon	Map3k5	protein_coding
10	19934469	19934679	184	216	222	265	130	193	0.58339736	4.155444293	13.40535095	0.000250908	0.004211658	+	Start codon	Map3k5	protein_coding
10	19934469	19934679	184	216	222	265	130	193	0.58339736	4.155444293	13.40535095	0.000250908	0.004211658	+	5'UTR	Map3k5	protein_coding
3	89773599	89773813	634	845	667	910	404	722	0.583397419	5.947380777	14.88757434	0.000114112	0.002354665	+	Exon	Ube2q1	protein_coding
3	89773599	89773813	634	845	667	910	404	722	0.583397419	5.947380777	14.88757434	0.000114112	0.002354665	+	Start codon	Ube2q1	protein_coding
3	89773599	89773813	634	845	667	910	404	722	0.583397419	5.947380777	14.88757434	0.000114112	0.002354665	+	5'UTR	Ube2q1	protein_coding
5	140830129	140830352	729	1142	470	920	461	718	0.584957964	6.047440011	14.35410386	0.000151449	0.002931458	-	Exon	Gna12	protein_coding
5	140830129	140830352	729	1142	470	920	461	718	0.584957964	6.047440011	14.35410386	0.000151449	0.002931458	-	Start codon	Gna12	protein_coding
5	140830129	140830352	729	1142	470	920	461	718	0.584957964	6.047440011	14.35410386	0.000151449	0.002931458	-	5'UTR	Gna12	protein_coding
5	110544347	110544550	464	560	382	556	325	419	0.585042458	5.336557216	15.40160934	8.69E-05	0.001905268	+	5'UTR	Galnt9	protein_coding
17	56009290	56009491	949	1063	585	1084	602	714	0.586235873	6.221305697	12.48237482	0.00041081	0.006082241	+	Exon	Mpnd	protein_coding
9	80067029	80067231	255	413	255	432	139	318	0.587005433	4.727017136	9.927387874	0.001628375	0.01666134	+	5'UTR	Senp6	protein_coding
11	97146460	97146777	1362	1987	1173	2055	935	1272	0.587377668	7.02227377	21.49581862	3.55E-06	0.00017882	-	Exon	Tbkbp1	protein_coding
11	97146460	97146777	1362	1987	1173	2055	935	1272	0.587377668	7.02227377	21.49581862	3.55E-06	0.00017882	-	3'UTR	Tbkbp1	protein_coding
1	52727213	52727413	302	375	223	326	194	304	0.588057337	4.687976815	11.44866706	0.000715453	0.009051007	-	5'UTR	Mfsd6	protein_coding
11	35853434	35853634	1022	1843	614	1636	640	876	0.588183094	6.607445242	16.09897969	6.01E-05	0.001474419	-	Exon	Wwc1	protein_coding
9	45955017	45955217	221	249	129	217	123	209	0.589505168	4.09793439	8.808469983	0.002998354	0.025460247	-	5'UTR	Sidt2	protein_coding
X	152144888	152145090	1529	1930	1026	2131	884	1262	0.590463287	7.012096032	16.22570036	5.62E-05	0.001414095	+	Exon	Iqsec2	protein_coding
9	22017993	22018193	591	770	549	982	430	438	0.591129412	5.788205023	12.71603923	0.000362533	0.005495819	-	3'UTR	Elavl3	protein_coding
7	122969971	122970181	1676	2113	1182	2032	1115	1446	0.5915927	7.160128068	16.63362771	4.53E-05	0.001204678	-	Exon	4930413G21Rik	TEC
7	19696604	19696804	31217	42203	22020	42652	19703	27163	0.591625014	11.4222717	19.89872656	8.17E-06	0.000347315	-	Exon	Apoe	protein_coding
17	34592291	34592708	389	603	265	512	248	386	0.591748038	5.158204412	14.96244619	0.000109672	0.002282106	+	Exon	Pbx2	protein_coding
17	34592291	34592708	389	603	265	512	248	386	0.591748038	5.158204412	14.96244619	0.000109672	0.002282106	+	Start codon	Pbx2	protein_coding
17	34592291	34592708	389	603	265	512	248	386	0.591748038	5.158204412	14.96244619	0.000109672	0.002282106	+	5'UTR	Pbx2	protein_coding
3	93445579	93445798	260	385	165	272	230	196	0.591825939	4.516174566	7.592343453	0.00586167	0.040915092	+	Exon	Tchh	protein_coding
19	5423625	5423836	4519	6849	4147	6871	3431	4227	0.591949197	8.804865447	21.54204127	3.46E-06	0.000176361	-	Exon	Drap1	protein_coding
9	107635143	107635344	6917	7944	4351	8245	4268	5421	0.592320216	9.113918354	14.54804558	0.00013663	0.002690147	-	Exon	Gnai2	protein_coding
9	107635143	107635344	6917	7944	4351	8245	4268	5421	0.592320216	9.113918354	14.54804558	0.00013663	0.002690147	-	Start codon	Gnai2	protein_coding
9	107635143	107635344	6917	7944	4351	8245	4268	5421	0.592320216	9.113918354	14.54804558	0.00013663	0.002690147	-	5'UTR	Gnai2	protein_coding
15	81586003	81586220	472	545	377	615	285	428	0.592594349	5.329960324	17.20891389	3.35E-05	0.000970607	+	5'UTR	Ep300	protein_coding
15	81586003	81586220	472	545	377	615	285	428	0.592594349	5.329960324	17.20891389	3.35E-05	0.000970607	+	Exon	Gm23880	miRNA
16	31664052	31664257	654	930	493	901	382	671	0.59310877	5.896270746	15.60985232	7.78E-05	0.001760752	+	5'UTR	Dlg1	protein_coding
3	94582339	94582544	1061	1172	1086	1628	727	859	0.593140299	6.584279912	16.87409058	3.99E-05	0.001105876	-	Exon	Snx27	protein_coding
1	74855024	74855247	3843	5058	2935	4962	2323	3831	0.593719645	8.413383404	17.54216812	2.81E-05	0.000859895	+	Exon	Cdk5r2	protein_coding
1	74855024	74855247	3843	5058	2935	4962	2323	3831	0.593719645	8.413383404	17.54216812	2.81E-05	0.000859895	+	Start codon	Cdk5r2	protein_coding
1	74855024	74855247	3843	5058	2935	4962	2323	3831	0.593719645	8.413383404	17.54216812	2.81E-05	0.000859895	+	5'UTR	Cdk5r2	protein_coding
11	74896870	74897078	1279	2260	937	2112	860	1177	0.593898505	6.990316096	20.06652038	7.48E-06	0.000321315	-	Exon	Sgsm2	protein_coding
11	74896870	74897078	1279	2260	937	2112	860	1177	0.593898505	6.990316096	20.06652038	7.48E-06	0.000321315	-	Start codon	Sgsm2	protein_coding
11	74896870	74897078	1279	2260	937	2112	860	1177	0.593898505	6.990316096	20.06652038	7.48E-06	0.000321315	-	5'UTR	Sgsm2	protein_coding
2	118923684	118923884	246	441	179	358	208	207	0.595919536	4.614743807	13.28551629	0.000267464	0.00440603	+	3'UTR	Bahd1	protein_coding
6	124841815	124842015	189	367	169	329	141	200	0.596351348	4.364426452	15.02979149	0.000105827	0.002225526	-	Exon	P3h3	protein_coding
6	124841815	124842015	189	367	169	329	141	200	0.596351348	4.364426452	15.02979149	0.000105827	0.002225526	-	3'UTR	P3h3	protein_coding
15	66286015	66286217	928	950	763	1132	575	804	0.596511695	6.255915513	18.75793282	1.48E-05	0.000529542	-	Exon	Kcnq3	protein_coding

11	94629810	94630012	508	569	329	602	302	422	0.597902184	5.342506004	14.13672922	0.000169992	0.003176184	+	Exon	Lrrc59	protein_coding
11	94629810	94630012	508	569	329	602	302	422	0.597902184	5.342506004	14.13672922	0.000169992	0.003176184	+	Start codon	Lrrc59	protein_coding
11	94629810	94630012	508	569	329	602	302	422	0.597902184	5.342506004	14.13672922	0.000169992	0.003176184	+	5'UTR	Lrrc59	protein_coding
2	118663335	118663535	140	179	108	173	95	132	0.598200763	3.621129322	12.17911931	0.000483275	0.006777445	+	5'UTR	Pak6	protein_coding
8	70597805	70598005	925	1319	806	1138	626	1054	0.598539906	6.456686093	13.90374059	0.000192415	0.003470495	-	Exon	Ssbp4	protein_coding
8	70597805	70598005	925	1319	806	1138	626	1054	0.598539906	6.456686093	13.90374059	0.000192415	0.003470495	-	Stop codon	Ssbp4	protein_coding
8	70597805	70598005	925	1319	806	1138	626	1054	0.598539906	6.456686093	13.90374059	0.000192415	0.003470495	-	3'UTR	Ssbp4	protein_coding
9	106886754	106886960	328	405	197	332	193	323	0.5991411	4.733906579	9.660843226	0.001882372	0.018388371	-	Exon	Rbm15b	protein_coding
13	93498929	93499129	252	211	253	293	158	224	0.60039864	4.358905911	15.52574226	8.14E-05	0.001816356	-	Exon	Jmy	protein_coding
16	91915884	91916084	95	217	133	216	89	119	0.601323925	3.678234278	9.521940785	0.002030298	0.019510405	+	3'UTR	Itsn1	protein_coding
3	8953718	8953921	1215	2007	820	1712	856	1106	0.601334266	6.846043194	19.49349669	1.01E-05	0.000397497	-	Exon	Tpd52	protein_coding
3	8953718	8953921	1215	2007	820	1712	856	1106	0.601334266	6.846043194	19.49349669	1.01E-05	0.000397497	-	Start codon	Tpd52	protein_coding
3	8953718	8953921	1215	2007	820	1712	856	1106	0.601334266	6.846043194	19.49349669	1.01E-05	0.000397497	-	5'UTR	Tpd52	protein_coding
3	8953718	8953921	1215	2007	820	1712	856	1106	0.601334266	6.846043194	19.49349669	1.01E-05	0.000397497	-	3'UTR	Tpd52	protein_coding
X	162643421	162643621	573	856	429	752	445	501	0.601983961	5.737369559	15.89649634	6.69E-05	0.001597256	-	Exon	Reps2	protein_coding
X	162643421	162643621	573	856	429	752	445	501	0.601983961	5.737369559	15.89649634	6.69E-05	0.001597256	-	Start codon	Reps2	protein_coding
X	162643421	162643621	573	856	429	752	445	501	0.601983961	5.737369559	15.89649634	6.69E-05	0.001597256	-	5'UTR	Reps2	protein_coding
15	101270503	101270704	1690	1691	1650	2527	982	1452	0.60204323	7.187992002	18.32249925	1.86E-05	0.000640029	+	Exon	Nr4a1	protein_coding
3	124385638	124385839	144	271	154	273	102	169	0.602552941	4.031435234	12.48122735	0.000411062	0.006082241	+	Exon	Gm4617	processed_pseudogene
1	82839540	82839751	703	888	462	711	387	786	0.603014449	5.881420539	8.678244026	0.003220311	0.026805094	+	Exon	Agfg1	protein_coding
1	82839540	82839751	703	888	462	711	387	786	0.603014449	5.881420539	8.678244026	0.003220311	0.026805094	+	Start codon	Agfg1	protein_coding
1	82839540	82839751	703	888	462	711	387	786	0.603014449	5.881420539	8.678244026	0.003220311	0.026805094	+	5'UTR	Agfg1	protein_coding
11	31671634	31671834	568	777	492	756	327	666	0.603708276	5.73006399	12.76129272	0.000353866	0.005397552	-	Exon	Bod1	protein_coding
11	31671634	31671834	568	777	492	756	327	666	0.603708276	5.73006399	12.76129272	0.000353866	0.005397552	-	Start codon	Bod1	protein_coding
11	31671634	31671834	568	777	492	756	327	666	0.603708276	5.73006399	12.76129272	0.000353866	0.005397552	-	5'UTR	Bod1	protein_coding
7	83883968	83884174	363	399	293	476	201	345	0.603955897	4.934389445	15.51673593	8.18E-05	0.001816356	-	5'UTR	Mesdc1	protein_coding
1	189343629	189343857	278	365	269	422	201	254	0.603982182	4.724334098	16.96692994	3.80E-05	0.001082801	-	5'UTR	Kcnk2	protein_coding
1	189343629	189343857	278	365	269	422	201	254	0.603982182	4.724334098	16.96692994	3.80E-05	0.001082801	+	Exon	A430027H14Rik	antisense
2	132263233	132263433	2798	3417	2342	3943	1906	2357	0.604230079	7.958761672	21.54283336	3.46E-06	0.000176361	+	5'UTR	Cds2	protein_coding
6	124931388	124931588	4666	7585	2908	6208	3167	4291	0.604794536	8.751104887	17.99398827	2.22E-05	0.00073501	+	5'UTR	Mlf2	protein_coding
12	81631378	81631679	750	919	624	1106	478	643	0.6053995	6.057758328	19.56435357	9.73E-06	0.000390808	+	Exon	Ttc9	protein_coding
12	81631378	81631679	750	919	624	1106	478	643	0.6053995	6.057758328	19.56435357	9.73E-06	0.000390808	+	Start codon	Ttc9	protein_coding
12	81631378	81631679	750	919	624	1106	478	643	0.6053995	6.057758328	19.56435357	9.73E-06	0.000390808	+	5'UTR	Ttc9	protein_coding
11	87127199	87127435	3543	3840	1986	4225	2097	2559	0.605441836	8.085781113	12.17339639	0.00048476	0.006786634	+	5'UTR	Trim37	protein_coding
19	29805497	29805996	970	1165	768	1228	591	947	0.605700759	6.395698958	17.99582567	2.21E-05	0.00073501	-	Exon	9930021J03Rik	protein_coding
19	29805497	29805996	970	1165	768	1228	591	947	0.605700759	6.395698958	17.99582567	2.21E-05	0.00073501	-	Start codon	9930021J03Rik	protein_coding
19	29805497	29805996	970	1165	768	1228	591	947	0.605700759	6.395698958	17.99582567	2.21E-05	0.00073501	-	5'UTR	9930021J03Rik	protein_coding
2	155826440	155826652	1514	2180	812	1977	934	1227	0.60580547	7.005849806	13.93509451	0.000189232	0.003438182	-	5'UTR	Eif6	protein_coding
14	105258741	105258942	577	1019	374	767	383	608	0.605850428	5.797171445	15.18364961	9.75E-05	0.002095932	+	Exon	Ndfip2	protein_coding
7	5059440	5059646	655	1248	444	1108	529	492	0.606786004	6.054127414	13.46187353	0.000243461	0.004134646	+	Exon	Ccdc106	protein_coding
7	5059440	5059646	655	1248	444	1108	529	492	0.606786004	6.054127414	13.46187353	0.000243461	0.004134646	+	Start codon	Ccdc106	protein_coding
7	5059440	5059646	655	1248	444	1108	529	492	0.606786004	6.054127414	13.46187353	0.000243461	0.004134646	+	5'UTR	Ccdc106	protein_coding
7	5059440	5059646	655	1248	444	1108	529	492	0.606786004	6.054127414	13.46187353	0.000243461	0.004134646	-	Exon	Gm45133	TEC
7	30956162	30956386	600	966	438	780	343	725	0.606799576	5.838196726	11.02155173	0.000900587	0.01075048	-	Exon	Usf2	protein_coding
11	97500305	97500516	2053	2274	2029	2850	1180	2171	0.606832728	7.529172299	17.42168394	2.99E-05	0.000895556	+	Exon	Arhgap23	protein_coding
11	97500305	97500516	2053	2274	2029	2850	1180	2171	0.606832728	7.529172299	17.42168394	2.99E-05	0.000895556	-	5'UTR	4933428G20Rik	protein_coding
11	97500305	97500516	2053	2274	2029	2850	1180	2171	0.606832728	7.529172299	17.42168394	2.99E-05	0.000895556	+	3'UTR	Arhgap23	protein_coding
7	92669540	92669740	383	574	210	472	194	403	0.607021268	5.048481242	9.709682454	0.001832997	0.018099242	-	Exon	Pcf11	protein_coding
7	92669540	92669740	383	574	210	472	194	403	0.607021268	5.048481242	9.709682454	0.001832997	0.018099242	-	Start codon	Pcf11	protein_coding
7	92669540	92669740	383	574	210	472	194	403	0.607021268	5.048481242	9.709682454	0.001832997	0.018099242	-	5'UTR	Pcf11	protein_coding
10	84440220	84440420	262	312	231	368	171	238	0.607045166	4.54569206	16.94759183	3.84E-05	0.001085276	-	Exon	Nuak1	protein_coding
10	84440220	84440420	262	312	231	368	171	238	0.607045166	4.54569206	16.94759183	3.84E-05	0.001085276	-	Start codon	Nuak1	protein_coding
10	84440220	84440420	262	312	231	368	171	238	0.607045166	4.54569206	16.94759183	3.84E-05	0.001085276	-	5'UTR	Nuak1	protein_coding
19	6979487	6979698	350	566	414	548	264	453	0.607050333	5.267406193	13.30383643	0.000264864	0.004370471	-	Intron	Fkbp2	protein_coding
14	55115020	55115223	6715	10043	4836	9500	4339	6423	0.607699653	9.280598994	21.53203352	3.48E-06	0.000176375	-	Exon	Jph4	protein_coding
1	191396484	191396684	296	427	341	449	207	366	0.608020621	4.947564441	13.91495782	0.00019127	0.003462482	-	5'UTR	Ppp2r5a	protein_coding
15	98834220	98834451	524	657	462	745	329	514	0.608055052	5.576237318	19.32645258	1.10E-05	0.00042493	-	3'UTR	Kmt2d	protein_coding
15	76666154	76666354	1986	3491	1495	3275	1551	1643	0.608764322	7.642390624	17.90874707	2.32E-05	0.000753504	+	Exon	Kifc2	protein_coding
8	107096172	107096372	301	393	247	415	203	280	0.609217449	4.769288608	17.54527094	2.81E-05	0.000859895	-	Exon	Terf2	protein_coding
15	80117728	80117928	2120	3527	1472	3150	1509	1904	0.609770962	7.670358017	21.61183255	3.34E-06	0.000173639	+	3'UTR	Syngr1	protein_coding
7	16273514	16273715	756	1045	833	1130	610	767	0.609824596	6.259885222	18.43546165	1.76E-05	0.000611682	-	5'UTR	Inafm1	protein_coding
7	44623076	44623283	482	591	267	617	267	391	0.610412766	5.272370538	12.76174868	0.00035378	0.005397552	-	Exon	Myh14	protein_coding
8	84415573	84415779	725	1177	417	1007	429	688	0.610708685	6.039617681	15.07852864	0.00010313	0.002178064	+	Exon	Cacna1a	protein_coding
8	84415573	84415779	725	1177	417	1007	429	688	0.610708685	6.039617681	15.07852864	0.00010313	0.002178064	+	Start codon	Cacna1a	protein_coding
8	84415573	84415779	725	1177	417	1007	429	688	0.610708685	6.039617681	15.07852864	0.00010313	0.002178064	+	5'UTR	Cacna1a	protein_coding
8	105637064	105637264	227	313	138	370	144	144	0.611814846	4.291983092	9.823483801	0.001722977	0.017306811	-	Exon	Gm5914	lincRNA
8	80879915	80880115	155	258	131	252	94	173	0.611950464	3.967822364	13.27513975	0.000268949	0.004423112	-	5'UTR	Gab1	protein_coding

8	80879915	80880115	155	258	131	252	94	173	0.611950464	3.967822364	13.27513975	0.000268949	0.004423112	+	Exon	RP24-335D17.3	antisense
4	115785015	115785219	733	1057	455	959	499	616	0.612247685	6.010811721	16.23535187	5.59E-05	0.001410498	+	Exon	Atpaf1	protein_coding
19	45024686	45024893	477	605	519	800	314	470	0.612571835	5.543454542	17.17163851	3.41E-05	0.000984069	+	Exon	Lzts2	protein_coding
18	37708959	37709159	342	242	355	338	208	322	0.612695695	4.741758062	14.34914913	0.000151848	0.002931458	+	Exon	Pcdhga6	protein_coding
10	127620784	127620988	752	822	1172	1272	627	727	0.613202708	6.301087179	12.9102302	0.000326791	0.005094642	-	5'UTR	Lrp1	protein_coding
11	5061363	5061595	651	1012	509	1018	457	589	0.613340959	5.971252352	20.7350519	5.27E-06	0.000254292	-	Exon	Gas2l1	protein_coding
18	38187187	38187469	1429	1984	1114	2151	998	1195	0.613613634	7.039747152	20.37263051	6.37E-06	0.000285048	-	3'UTR	Pcdh1	protein_coding
3	108186402	108186604	432	510	404	513	287	476	0.614845829	5.290478756	14.63514759	0.000130459	0.002608283	+	5'UTR	Amigo1	protein_coding
9	77754648	77754848	491	545	293	597	265	417	0.615032796	5.269964385	13.15147456	0.000287293	0.004624763	+	Exon	Gclc	protein_coding
9	77754648	77754848	491	545	293	597	265	417	0.615032796	5.269964385	13.15147456	0.000287293	0.004624763	+	Start codon	Gclc	protein_coding
9	77754648	77754848	491	545	293	597	265	417	0.615032796	5.269964385	13.15147456	0.000287293	0.004624763	+	5'UTR	Gclc	protein_coding
5	138995115	138995339	778	969	576	864	500	802	0.615171039	6.074021619	13.57412498	0.000229325	0.003968756	+	Exon	6330403L08Rik	antisense
7	28268084	28268293	1459	1715	1346	1703	753	1895	0.615517396	7.040401349	8.71291461	0.003159636	0.02637656	+	Exon	Eid2	protein_coding
7	127707504	127707704	192	252	74	248	107	126	0.61581002	3.883085991	8.108321067	0.004406252	0.033347162	-	Exon	Bcl7c	protein_coding
6	71440134	71440401	1061	1358	884	1380	630	1162	0.616480839	6.586848677	15.83911677	6.90E-05	0.001634582	-	Exon	Rmnd5a	protein_coding
6	71440134	71440401	1061	1358	884	1380	630	1162	0.616480839	6.586848677	15.83911677	6.90E-05	0.001634582	-	Start codon	Rmnd5a	protein_coding
6	71440134	71440401	1061	1358	884	1380	630	1162	0.616480839	6.586848677	15.83911677	6.90E-05	0.001634582	-	5'UTR	Rmnd5a	protein_coding
15	98763022	98763224	3468	4751	2272	4639	2202	3067	0.617067969	8.246600476	19.11943732	1.23E-05	0.000461459	-	5'UTR	Arf3	protein_coding
11	120632573	120632789	185	293	134	237	102	231	0.617810147	4.132652009	9.756131262	0.001787256	0.017754011	-	Intron	Mafg	protein_coding
8	33599722	33599923	2176	2967	1301	2696	1214	2135	0.617926177	7.536849566	13.4339416	0.000247112	0.004168018	+	Exon	Ppp2cb	protein_coding
8	33599722	33599923	2176	2967	1301	2696	1214	2135	0.617926177	7.536849566	13.4339416	0.000247112	0.004168018	+	Start codon	Ppp2cb	protein_coding
8	33599722	33599923	2176	2967	1301	2696	1214	2135	0.617926177	7.536849566	13.4339416	0.000247112	0.004168018	+	5'UTR	Ppp2cb	protein_coding
11	120624668	120624875	353	671	285	507	301	369	0.618384348	5.222665549	16.23819914	5.59E-05	0.001410498	-	Exon	Sirt7	protein_coding
11	120624668	120624875	353	671	285	507	301	369	0.618384348	5.222665549	16.23819914	5.59E-05	0.001410498	-	Exon	Mir6936	miRNA
14	34819973	34820173	269	435	288	416	200	332	0.61928933	4.848640503	14.97692194	0.000108834	0.00227356	+	5'UTR	Grid1	protein_coding
11	97187722	97187924	262	261	324	318	164	326	0.620537986	4.612607179	11.97213205	0.000540021	0.007341911	-	5'UTR	Kpnb1	protein_coding
2	153529573	153529773	248	406	311	436	174	335	0.62073692	4.814114583	12.17856753	0.000483418	0.006777445	-	Exon	Nol4l	protein_coding
2	153529573	153529773	248	406	311	436	174	335	0.62073692	4.814114583	12.17856753	0.000483418	0.006777445	-	Start codon	Nol4l	protein_coding
2	153529573	153529773	248	406	311	436	174	335	0.62073692	4.814114583	12.17856753	0.000483418	0.006777445	-	5'UTR	Nol4l	protein_coding
11	102393467	102393677	1918	3254	1296	3003	1306	1741	0.621430736	7.535771514	22.02125291	2.70E-06	0.000148894	+	Exon	Rundc3a	protein_coding
11	102393467	102393677	1918	3254	1296	3003	1306	1741	0.621430736	7.535771514	22.02125291	2.70E-06	0.000148894	+	Start codon	Rundc3a	protein_coding
11	102393467	102393677	1918	3254	1296	3003	1306	1741	0.621430736	7.535771514	22.02125291	2.70E-06	0.000148894	+	5'UTR	Rundc3a	protein_coding
3	146404685	146404885	1420	1869	758	1806	933	1040	0.622789429	6.869563102	12.75427417	0.000355197	0.005409496	+	5'UTR	Ssx2ip	protein_coding
19	4396632	4396906	198	307	189	250	146	253	0.623662776	4.329736206	11.53270767	0.000683823	0.008789221	-	5'UTR	Kdm2a	protein_coding
19	59943306	59943552	664	871	538	827	479	649	0.624313871	5.917281755	18.04029852	2.16E-05	0.000729587	-	5'UTR	Rab11fip2	protein_coding
13	9276556	9276758	631	663	538	852	350	609	0.624657825	5.744223767	17.91050058	2.32E-05	0.000753504	+	5'UTR	Dip2c	protein_coding
7	45700227	45700448	821	1613	870	1516	756	851	0.624788862	6.582494556	16.61807509	4.57E-05	0.001207499	+	Exon	Car11	protein_coding
7	45700227	45700448	821	1613	870	1516	756	851	0.624788862	6.582494556	16.61807509	4.57E-05	0.001207499	+	Start codon	Car11	protein_coding
7	45700227	45700448	821	1613	870	1516	756	851	0.624788862	6.582494556	16.61807509	4.57E-05	0.001207499	+	5'UTR	Car11	protein_coding
9	104062891	104063093	493	917	250	690	311	466	0.625084549	5.536801139	14.63724124	0.000130314	0.002608283	-	Exon	Uba5	protein_coding
9	104062891	104063093	493	917	250	690	311	466	0.625084549	5.536801139	14.63724124	0.000130314	0.002608283	-	Start codon	Uba5	protein_coding
9	104062891	104063093	493	917	250	690	311	466	0.625084549	5.536801139	14.63724124	0.000130314	0.002608283	-	5'UTR	Uba5	protein_coding
10	80640364	80640564	767	1270	722	1370	534	796	0.625406569	6.328520478	19.74965125	8.83E-06	0.000363557	+	3'UTR	Csnk1g2	protein_coding
2	25561760	25561960	877	1527	731	1329	780	780	0.62563188	6.501923343	16.46255269	4.96E-05	0.001280547	+	Exon	Edf1	protein_coding
2	25561760	25561960	877	1527	731	1329	780	780	0.62563188	6.501923343	16.46255269	4.96E-05	0.001280547	+	Stop codon	Edf1	protein_coding
2	25561760	25561960	877	1527	731	1329	780	780	0.62563188	6.501923343	16.46255269	4.96E-05	0.001280547	+	3'UTR	Edf1	protein_coding
5	88565035	88565240	282	349	174	344	152	275	0.625705776	4.545704385	12.49770037	0.000407453	0.006046949	+	Exon	Rufy3	protein_coding
5	88565035	88565240	282	349	174	344	152	275	0.625705776	4.545704385	12.49770037	0.000407453	0.006046949	+	Start codon	Rufy3	protein_coding
5	88565035	88565240	282	349	174	344	152	275	0.625705776	4.545704385	12.49770037	0.000407453	0.006046949	+	5'UTR	Rufy3	protein_coding
4	155668850	155669060	277	460	239	372	240	286	0.626850438	4.81834668	14.71327356	0.000125162	0.002529861	-	5'UTR	Mib2	protein_coding
19	6975171	6975371	821	938	563	948	474	804	0.627738368	6.08363063	14.53807631	0.000137355	0.002699043	+	Exon	Ppp1r14b	protein_coding
19	6975171	6975371	821	938	563	948	474	804	0.627738368	6.08363063	14.53807631	0.000137355	0.002699043	+	Start codon	Ppp1r14b	protein_coding
19	6975171	6975371	821	938	563	948	474	804	0.627738368	6.08363063	14.53807631	0.000137355	0.002699043	+	5'UTR	Ppp1r14b	protein_coding
19	18670882	18671082	243	343	156	273	128	292	0.628580247	4.41787603	8.799703074	0.003012796	0.025542119	+	Exon	Carnmt1	protein_coding
19	18670882	18671082	243	343	156	273	128	292	0.628580247	4.41787603	8.799703074	0.003012796	0.025542119	+	Start codon	Carnmt1	protein_coding
19	18670882	18671082	243	343	156	273	128	292	0.628580247	4.41787603	8.799703074	0.003012796	0.025542119	+	5'UTR	Carnmt1	protein_coding
10	80399226	80399428	2867	3131	3722	4575	2234	2680	0.629305317	8.147604927	18.27037111	1.92E-05	0.000655508	-	Exon	Mbd3	protein_coding
10	80399226	80399428	2867	3131	3722	4575	2234	2680	0.629305317	8.147604927	18.27037111	1.92E-05	0.000655508	-	Start codon	Mbd3	protein_coding
10	80399226	80399428	2867	3131	3722	4575	2234	2680	0.629305317	8.147604927	18.27037111	1.92E-05	0.000655508	-	5'UTR	Mbd3	protein_coding
1	17097591	17097791	278	325	244	346	162	315	0.629610347	4.630120079	13.65146834	0.000220069	0.003836267	-	Exon	Jph1	protein_coding
1	17097591	17097791	278	325	244	346	162	315	0.629610347	4.630120079	13.65146834	0.000220069	0.003836267	-	Start codon	Jph1	protein_coding
1	17097591	17097791	278	325	244	346	162	315	0.629610347	4.630120079	13.65146834	0.000220069	0.003836267	-	5'UTR	Jph1	protein_coding
2	90917553	90917753	322	531	185	439	225	283	0.629779343	4.88786917	15.56112979	7.99E-05	0.001798483	-	Exon	Ptpmt1	processed_transcript
2	90917553	90917753	322	531	185	439	225	283	0.629779343	4.88786917	15.56112979	7.99E-05	0.001798483	+	Exon	Gm17661	pseudogene
14	31001656	31001881	283	359	171	373	163	249	0.63319181	4.563891291	14.51142545	0.000139312	0.002732064	-	5'UTR	Spcs1	protein_coding
14	31001656	31001881	283	359	171	373	163	249	0.63319181	4.563891291	14.51142545	0.000139312	0.002732064	+	5'UTR	Glt8d1	protein_coding
9	44696119	44696324	1670	2284	1117	2520	959	1505	0.633675159	7.210059961	17.93114158	2.29E-05	0.000753504	-	Exon	Phldb1	protein_coding

8	70698975	70699175	1592	2307	1234	2114	974	1818	0.634137721	7.224956882	15.74412015	7.25E-05	0.001682437	+	Exon	Jund	protein_coding
8	70698975	70699175	1592	2307	1234	2114	974	1818	0.634137721	7.224956882	15.74412015	7.25E-05	0.001682437	+	Start codon	Jund	protein_coding
8	70698975	70699175	1592	2307	1234	2114	974	1818	0.634137721	7.224956882	15.74412015	7.25E-05	0.001682437	+	5'UTR	Jund	protein_coding
8	70698975	70699175	1592	2307	1234	2114	974	1818	0.634137721	7.224956882	15.74412015	7.25E-05	0.001682437	-	Exon	Gm11175	antisense
12	81493546	81493747	306	412	249	402	229	292	0.635563721	4.821621986	17.62483566	2.69E-05	0.000836635	+	Exon	Gm28370	antisense
12	81493546	81493747	306	412	249	402	229	292	0.635563721	4.821621986	17.62483566	2.69E-05	0.000836635	-	Intron	Gm20498	protein_coding
5	144100403	144100603	266	341	243	341	203	270	0.635792691	4.638442061	16.58160341	4.66E-05	0.001221248	+	5'UTR	Lmtk2	protein_coding
11	100970213	100970413	279	352	240	393	149	321	0.636358788	4.674711273	13.24492945	0.000273318	0.004465251	-	Exon	Ptrf	protein_coding
13	103752561	103752770	1891	1520	1403	2172	928	1684	0.638159231	7.144308762	14.79742156	0.000119699	0.002429375	-	Exon	Srek1	protein_coding
13	103752561	103752770	1891	1520	1403	2172	928	1684	0.638159231	7.144308762	14.79742156	0.000119699	0.002429375	-	3'UTR	Srek1	protein_coding
7	19176451	19176654	512	735	395	790	365	442	0.638439318	5.586612893	20.34742984	6.46E-06	0.000287525	+	Exon	Eml2	protein_coding
7	19176451	19176654	512	735	395	790	365	442	0.638439318	5.586612893	20.34742984	6.46E-06	0.000287525	+	Start codon	Eml2	protein_coding
7	19176451	19176654	512	735	395	790	365	442	0.638439318	5.586612893	20.34742984	6.46E-06	0.000287525	+	5'UTR	Eml2	protein_coding
11	96065108	96065328	547	790	262	667	303	511	0.638821737	5.517820605	11.44051825	0.000718597	0.009071031	-	Exon	Ube2z	protein_coding
11	96065108	96065328	547	790	262	667	303	511	0.638821737	5.517820605	11.44051825	0.000718597	0.009071031	-	Start codon	Ube2z	protein_coding
11	96065108	96065328	547	790	262	667	303	511	0.638821737	5.517820605	11.44051825	0.000718597	0.009071031	-	5'UTR	Ube2z	protein_coding
17	37048470	37048692	1190	2163	839	1891	878	1139	0.638871009	6.914269779	24.26062339	8.41E-07	6.03E-05	+	Exon	Gabbr1	protein_coding
4	149426203	149426403	465	673	373	697	259	531	0.639851092	5.465652983	14.66071038	0.000128701	0.0025908	-	5'UTR	Ube4b	protein_coding
5	20702307	20702507	683	824	513	890	346	761	0.640057186	5.89062098	12.46607421	0.00041441	0.006113477	+	Exon	Magi2	protein_coding
5	20702307	20702507	683	824	513	890	346	761	0.640057186	5.89062098	12.46607421	0.00041441	0.006113477	+	3'UTR	Magi2	protein_coding
5	20702307	20702507	683	824	513	890	346	761	0.640057186	5.89062098	12.46607421	0.00041441	0.006113477	+	Exon	Gm29254	3prime_overlapping_ncRNA
2	150904457	150904693	3344	4443	1955	4258	2125	2870	0.6409579	8.150259243	16.77974171	4.20E-05	0.001139893	-	Exon	Abhd12	protein_coding
2	150904457	150904693	3344	4443	1955	4258	2125	2870	0.6409579	8.150259243	16.77974171	4.20E-05	0.001139893	-	Start codon	Abhd12	protein_coding
2	150904457	150904693	3344	4443	1955	4258	2125	2870	0.6409579	8.150259243	16.77974171	4.20E-05	0.001139893	-	5'UTR	Abhd12	protein_coding
17	35988457	35988657	3012	4436	1912	4382	2060	2499	0.641474952	8.091119796	19.81601353	8.53E-06	0.00035262	+	Exon	Gnl1	protein_coding
17	25013927	25014127	198	213	184	218	117	242	0.64297493	4.12561482	10.96725646	0.00092736	0.011003628	-	Exon	Cramp1l	protein_coding
17	25013927	25014127	198	213	184	218	117	242	0.64297493	4.12561482	10.96725646	0.00092736	0.011003628	-	Start codon	Cramp1l	protein_coding
17	25013927	25014127	198	213	184	218	117	242	0.64297493	4.12561482	10.96725646	0.00092736	0.011003628	-	5'UTR	Cramp1l	protein_coding
8	122475891	122476091	238	394	162	404	136	233	0.643503409	4.522612113	15.05164032	0.000104609	0.002204602	-	Exon	Rnf166	protein_coding
8	122475891	122476091	238	394	162	404	136	233	0.643503409	4.522612113	15.05164032	0.000104609	0.002204602	-	Start codon	Rnf166	protein_coding
8	122475891	122476091	238	394	162	404	136	233	0.643503409	4.522612113	15.05164032	0.000104609	0.002204602	-	5'UTR	Rnf166	protein_coding
6	86733039	86733239	243	317	194	329	157	250	0.645155932	4.468246682	17.25215074	3.27E-05	0.000954365	-	Exon	Gmcl1	protein_coding
6	86733039	86733239	243	317	194	329	157	250	0.645155932	4.468246682	17.25215074	3.27E-05	0.000954365	-	Start codon	Gmcl1	protein_coding
6	86733039	86733239	243	317	194	329	157	250	0.645155932	4.468246682	17.25215074	3.27E-05	0.000954365	-	5'UTR	Gmcl1	protein_coding
7	27658925	27659125	1249	1901	932	1800	911	1185	0.645312197	6.898910844	23.75857941	1.09E-06	7.60E-05	-	Exon	Map3k10	protein_coding
7	19286537	19286748	425	519	363	596	271	426	0.646552205	5.268011399	20.64170583	5.54E-06	0.000259641	+	Exon	Rtn2	protein_coding
10	40349436	40349639	1199	957	1245	1586	749	1009	0.647047287	6.632572407	24.23208955	8.54E-07	6.03E-05	-	Exon	Cdk19os	antisense
10	40349436	40349639	1199	957	1245	1586	749	1009	0.647047287	6.632572407	24.23208955	8.54E-07	6.03E-05	+	Exon	Cdk19	protein_coding
10	40349436	40349639	1199	957	1245	1586	749	1009	0.647047287	6.632572407	24.23208955	8.54E-07	6.03E-05	+	Start codon	Cdk19	protein_coding
10	40349436	40349639	1199	957	1245	1586	749	1009	0.647047287	6.632572407	24.23208955	8.54E-07	6.03E-05	+	5'UTR	Cdk19	protein_coding
9	60724023	60724246	727	1030	425	946	471	649	0.647836815	5.987814161	16.94317365	3.85E-05	0.001085276	+	Exon	Larp6	protein_coding
7	16614522	16614763	466	451	621	627	314	554	0.647995853	5.48493502	16.70745223	4.36E-05	0.001174482	-	5'UTR	Arhgap35	protein_coding
7	16614522	16614763	466	451	621	627	314	554	0.647995853	5.48493502	16.70745223	4.36E-05	0.001174482	+	Intron	Gm29443	antisense
1	106171925	106172125	193	285	306	356	132	302	0.648740197	4.529346394	9.435072222	0.002128747	0.02015377	+	Exon	Phlpp1	protein_coding
1	106171925	106172125	193	285	306	356	132	302	0.648740197	4.529346394	9.435072222	0.002128747	0.02015377	+	Start codon	Phlpp1	protein_coding
1	106171925	106172125	193	285	306	356	132	302	0.648740197	4.529346394	9.435072222	0.002128747	0.02015377	+	5'UTR	Phlpp1	protein_coding
X	7573980	7574188	265	344	233	345	191	281	0.649758849	4.631961989	17.46050533	2.93E-05	0.000883951	-	Exon	Ppp1r3f	protein_coding
1	75422005	75422215	333	520	227	451	180	403	0.650007713	4.97003245	11.66682218	0.000636246	0.00835316	+	Exon	Speg	protein_coding
7	25237522	25237725	6457	8122	4862	8420	4160	6378	0.650854038	9.165416991	22.10683069	2.58E-06	0.000143911	-	Exon	Gsk3a	protein_coding
7	25237522	25237725	6457	8122	4862	8420	4160	6378	0.650854038	9.165416991	22.10683069	2.58E-06	0.000143911	-	Start codon	Gsk3a	protein_coding
7	25237522	25237725	6457	8122	4862	8420	4160	6378	0.650854038	9.165416991	22.10683069	2.58E-06	0.000143911	-	5'UTR	Gsk3a	protein_coding
7	45685667	45685940	213	221	206	323	150	169	0.651078022	4.239070799	16.05871812	6.14E-05	0.001494973	+	Intron	Ntn5	protein_coding
7	45685667	45685940	213	221	206	323	150	169	0.651078022	4.239070799	16.05871812	6.14E-05	0.001494973	-	Intron	Sec1	protein_coding
6	83122137	83122371	496	711	439	755	370	488	0.651429579	5.601010602	23.15308807	1.50E-06	9.48E-05	-	Exon	Ino80b	protein_coding
6	83122137	83122371	496	711	439	755	370	488	0.651429579	5.601010602	23.15308807	1.50E-06	9.48E-05	-	3'UTR	Ino80b	protein_coding
9	72532352	72532559	558	646	372	583	367	547	0.65247025	5.534658206	13.13896918	0.000289217	0.004648163	+	5'UTR	Rfx7	protein_coding
17	37045998	37046219	1494	2256	1341	2160	1174	1567	0.652487252	7.229597969	23.70626349	1.12E-06	7.66E-05	+	5'UTR	Gabbr1	protein_coding
7	80371115	80371316	254	386	213	379	151	308	0.653180113	4.644975367	14.61619212	0.000131777	0.0026207	-	5'UTR	Man2a2	protein_coding
4	33924637	33924844	395	446	496	492	293	508	0.653199296	5.296890742	13.54320611	0.000233134	0.004007469	+	5'UTR	Cnr1	protein_coding
2	25441788	25442021	680	1246	401	1014	442	692	0.653585846	6.055487111	18.91612275	1.37E-05	0.000500033	+	Exon	Abca2	protein_coding
4	154160461	154160661	2302	3289	1392	3213	1420	2107	0.653777872	7.67342782	19.07645455	1.26E-05	0.000470182	-	Exon	Tprgl	protein_coding
4	154160461	154160661	2302	3289	1392	3213	1420	2107	0.653777872	7.67342782	19.07645455	1.26E-05	0.000470182	-	Start codon	Tprgl	protein_coding
4	154160461	154160661	2302	3289	1392	3213	1420	2107	0.653777872	7.67342782	19.07645455	1.26E-05	0.000470182	-	5'UTR	Tprgl	protein_coding
4	93335167	93335373	300	397	180	350	187	297	0.654482546	4.678353364	13.64484328	0.000220847	0.003843049	-	Exon	Tusc1	protein_coding
5	138263441	138263738	439	910	259	604	340	464	0.656558579	5.502169263	16.78064111	4.20E-05	0.001139893	-	Exon	BC037034	protein_coding
2	131262519	131262719	491	634	315	520	325	510	0.657509361	5.398878164	12.41725851	0.000425384	0.006210484	+	Exon	Pank2	protein_coding
2	131262519	131262719	491	634	315	520	325	510	0.657509361	5.398878164	12.41725851	0.000425384	0.006210484	+	Start codon	Pank2	protein_coding

2	131262519	131262719	491	634	315	520	325	510	0.657509361	5.398878164	12.41725851	0.000425384	0.006210484	+	5'UTR	Pank2	protein_coding
X	20291513	20291721	366	426	339	506	246	373	0.657970875	5.066341915	21.26504915	4.00E-06	0.000198652	-	Exon	Slc9a7	protein_coding
X	20291513	20291721	366	426	339	506	246	373	0.657970875	5.066341915	21.26504915	4.00E-06	0.000198652	-	Start codon	Slc9a7	protein_coding
X	20291513	20291721	366	426	339	506	246	373	0.657970875	5.066341915	21.26504915	4.00E-06	0.000198652	-	5'UTR	Slc9a7	protein_coding
11	69340759	69340963	824	1256	496	1152	608	674	0.658770834	6.230721102	17.62398324	2.69E-05	0.000836635	+	Exon	Chd3os	protein_coding
11	101468162	101468362	908	943	668	1164	550	802	0.659719517	6.225682763	20.57536959	5.73E-06	0.000267283	+	Exon	Rnd2	protein_coding
11	101468162	101468362	908	943	668	1164	550	802	0.659719517	6.225682763	20.57536959	5.73E-06	0.000267283	+	Start codon	Rnd2	protein_coding
11	101468162	101468362	908	943	668	1164	550	802	0.659719517	6.225682763	20.57536959	5.73E-06	0.000267283	+	5'UTR	Rnd2	protein_coding
1	42696744	42696944	611	750	472	759	330	725	0.660436059	5.760836505	12.58701676	0.000388436	0.00584368	+	5'UTR	Pou3f3	protein_coding
3	38885930	38886130	161	110	201	237	121	97	0.662155902	3.758524483	10.09044777	0.001490399	0.015506424	-	Intron	C230034O21Rik	antisense
2	49619368	49619596	2853	3871	1879	3879	1624	2961	0.662840672	7.986456953	16.96612771	3.81E-05	0.001082801	+	5'UTR	Kif5c	protein_coding
6	117716505	117716776	67	70	41	57	43	71	0.663901921	2.39930952	7.57765144	0.005909639	0.04107656	+	Exon	Gm7292	TEC
6	117716505	117716776	67	70	41	57	43	71	0.663901921	2.39930952	7.57765144	0.005909639	0.04107656	-	Intron	1700030F04Rik	lincRNA
9	106465156	106465363	303	386	253	427	213	294	0.664102016	4.803774711	20.89974133	4.84E-06	0.000235638	-	Exon	Gpr62	protein_coding
3	107517731	107517939	1030	1488	731	1431	714	1004	0.664527684	6.581216497	22.98755733	1.63E-06	9.94E-05	-	5'UTR	Slc6a17	protein_coding
7	18958235	18958445	1463	2006	1340	2071	1071	1592	0.666358484	7.159889716	24.47013083	7.55E-07	5.61E-05	+	Exon	Nova2	protein_coding
9	118926573	118926791	269	255	230	284	158	300	0.666391594	4.480760057	13.01384902	0.000309196	0.004866387	+	5'UTR	Ctdspl	protein_coding
4	121016881	121017095	4090	6347	3290	5834	2783	4724	0.667556473	8.662441879	21.19778386	4.14E-06	0.000204719	-	5'UTR	Smap2	protein_coding
15	81585452	81585760	173	228	230	290	88	273	0.667708113	4.230438517	7.984365721	0.004718302	0.034814254	+	5'UTR	Ep300	protein_coding
17	56613765	56613973	4725	7914	2907	7275	3235	4344	0.667954001	8.822960529	23.38817943	1.32E-06	8.61E-05	+	Exon	Rpl36	protein_coding
4	152128928	152129135	435	329	417	582	281	317	0.668462319	5.117996366	18.00089087	2.21E-05	0.00073501	-	Intron	Espn	protein_coding
14	55109472	55109694	2544	4121	2023	4005	1846	2642	0.669616987	8.002472859	28.56784491	9.05E-08	1.04E-05	-	Exon	Jph4	protein_coding
10	80797933	80798133	246	305	178	460	116	204	0.671730481	4.448282641	10.3751238	0.001277246	0.013842432	-	Exon	Plekhj1	protein_coding
19	10422720	10422941	654	1110	347	958	413	616	0.672174036	5.928548022	17.71375368	2.57E-05	0.000808232	+	Intron	Syt7	protein_coding
19	42268313	42268514	922	1375	561	1300	648	803	0.672847551	6.391432891	20.16649444	7.10E-06	0.000310452	+	Exon	Golga7b	protein_coding
19	42268313	42268514	922	1375	561	1300	648	803	0.672847551	6.391432891	20.16649444	7.10E-06	0.000310452	+	Stop codon	Golga7b	protein_coding
19	42268313	42268514	922	1375	561	1300	648	803	0.672847551	6.391432891	20.16649444	7.10E-06	0.000310452	+	3'UTR	Golga7b	protein_coding
5	113138284	113138552	262	259	244	351	170	250	0.67287564	4.507547211	20.45915063	6.09E-06	0.000279209	-	Intron	2900026A02Rik	protein_coding
6	23838951	23839152	740	907	507	947	530	632	0.673390101	6.001426892	19.27417453	1.13E-05	0.000433776	-	Exon	Cadps2	protein_coding
6	23838951	23839152	740	907	507	947	530	632	0.673390101	6.001426892	19.27417453	1.13E-05	0.000433776	-	Start codon	Cadps2	protein_coding
6	23838951	23839152	740	907	507	947	530	632	0.673390101	6.001426892	19.27417453	1.13E-05	0.000433776	-	5'UTR	Cadps2	protein_coding
16	20715990	20716196	366	393	225	477	225	287	0.673917908	4.873133959	16.09920284	6.01E-05	0.001474419	-	Exon	Clcn2	protein_coding
16	20715990	20716196	366	393	225	477	225	287	0.673917908	4.873133959	16.09920284	6.01E-05	0.001474419	-	Start codon	Clcn2	protein_coding
16	20715990	20716196	366	393	225	477	225	287	0.673917908	4.873133959	16.09920284	6.01E-05	0.001474419	-	5'UTR	Clcn2	protein_coding
7	117381230	117381431	476	502	489	555	368	507	0.674601994	5.447656323	17.43247188	2.98E-05	0.000894357	+	Exon	Xylt1	protein_coding
8	111258865	111259095	1490	1969	842	1870	990	1287	0.675157588	6.988278966	16.89740437	3.95E-05	0.00109853	-	Exon	Glg1	protein_coding
10	60831222	60831439	420	580	312	612	243	461	0.67552653	5.282049625	17.50682428	2.86E-05	0.000873325	-	5'UTR	Unc5b	protein_coding
17	10318901	10319131	2556	2653	2574	3368	1386	3169	0.67558395	7.860438762	13.99635016	0.000183166	0.003358833	-	5'UTR	Qk	protein_coding
7	29019987	29020296	153	230	128	250	105	157	0.675688401	3.920135532	17.75991592	2.51E-05	0.000793463	-	Exon	Ryr1	protein_coding
7	19196416	19196618	481	815	319	846	344	400	0.677324275	5.566392818	18.96072015	1.33E-05	0.000491671	+	Exon	Eml2	protein_coding
16	15887602	15887802	327	395	229	423	150	392	0.678196909	4.822954878	10.2656911	0.00135527	0.014403747	+	Exon	Cebpd	protein_coding
2	32535313	32535549	500	819	298	579	286	642	0.67948829	5.550722864	9.71376279	0.001828932	0.01807716	+	5'UTR	Fam102a	protein_coding
10	81325006	81325206	214	385	119	318	133	224	0.68014484	4.370050085	16.00696483	6.31E-05	0.00152141	+	Exon	Cactin	protein_coding
10	13966214	13966414	240	445	346	488	205	356	0.680464885	4.944660495	13.26880953	0.000269859	0.0044307	+	5'UTR	Hivep2	protein_coding
12	59061251	59061451	1582	2969	1280	2661	1219	1761	0.681687121	7.420936071	28.50008954	9.37E-08	1.06E-05	-	5'UTR	Trappc6b	protein_coding
1	180726103	180726303	385	308	287	429	195	371	0.682769508	4.869868096	14.85776077	0.00011593	0.002372366	+	Exon	Acbd3	protein_coding
1	180726103	180726303	385	308	287	429	195	371	0.682769508	4.869868096	14.85776077	0.00011593	0.002372366	+	Start codon	Acbd3	protein_coding
17	84186756	84186956	328	393	225	387	228	313	0.683489689	4.816318212	16.87892608	3.98E-05	0.001105876	-	Exon	Zfp36l2	protein_coding
X	8132635	8132840	438	782	471	889	304	551	0.685146136	5.657867581	16.69267349	4.40E-05	0.001180451	-	5'UTR	Wdr13	protein_coding
4	4792839	4793042	1319	1673	612	1513	762	1194	0.685707357	6.730172736	12.03974873	0.00052078	0.007159094	-	Exon	Impad1	protein_coding
2	173777942	173778155	1140	1965	679	1722	749	1151	0.68605568	6.785746309	21.62247257	3.32E-06	0.000173592	+	Exon	Vapb	protein_coding
11	100957727	100957927	116	291	149	247	92	208	0.686317274	4.03080914	10.12209456	0.001465024	0.01530687	-	3'UTR	Ptrf	protein_coding
8	93958906	93959129	2228	3872	1432	3366	1404	2492	0.687022397	7.783223197	19.85205765	8.37E-06	0.000348954	+	3'UTR	Gnao1	protein_coding
1	59633030	59633232	250	320	209	394	187	212	0.687169986	4.544417898	19.5204938	9.95E-06	0.000397497	+	Exon	Gm973	protein_coding
5	102069740	102069940	756	426	991	893	374	812	0.687247135	5.94783459	17.41267062	3.01E-05	0.000895556	-	5'UTR	Wdfy3	protein_coding
8	11556635	11556835	172	221	112	217	112	171	0.68905771	3.90814101	15.71844968	7.35E-05	0.00168697	+	5'UTR	Ing1	protein_coding
4	43562231	43562431	204	321	203	327	103	317	0.689091536	4.443157357	8.778278554	0.003048387	0.025708415	-	5'UTR	Tln1	protein_coding
4	43562231	43562431	204	321	203	327	103	317	0.689091536	4.443157357	8.778278554	0.003048387	0.025708415	+	5'UTR	Creb3	protein_coding
7	80370890	80371100	276	328	140	357	159	229	0.691592002	4.467384742	14.06150373	0.000176928	0.00326261	-	5'UTR	Man2a2	protein_coding
15	84987744	84987944	887	1141	750	1326	480	1068	0.69164641	6.383175002	15.28825353	9.23E-05	0.002004793	-	5'UTR	5031439G07Rik	protein_coding
15	84987744	84987944	887	1141	750	1326	480	1068	0.69164641	6.383175002	15.28825353	9.23E-05	0.002004793	-	Exon	5031439G07Rik	protein_coding
15	84987744	84987944	887	1141	750	1326	480	1068	0.69164641	6.383175002	15.28825353	9.23E-05	0.002004793	-	Start codon	5031439G07Rik	protein_coding
5	124863143	124863366	154	252	150	281	127	156	0.693255743	4.05420951	18.49867391	1.70E-05	0.000595923	+	5'UTR	Zfp664	protein_coding
1	172274084	172274284	9678	10736	7020	12863	5659	9582	0.693987628	9.692388056	21.58863578	3.38E-06	0.000174832	-	3'UTR	Atp1a2	protein_coding
7	16278451	16278657	175	344	147	299	141	206	0.694251988	4.290200902	19.89359355	8.19E-06	0.000347315	-	Exon	Ccdc9	protein_coding
9	57962257	57962483	253	486	232	510	171	305	0.694292183	4.84703116	17.75490641	2.51E-05	0.000793463	+	3'UTR	Sema7a	protein_coding
3	88200747	88200999	808	906	600	1010	499	833	0.696724727	6.122418492	20.06187096	7.50E-06	0.000321315	-	Exon	AI849053	lincRNA

3	88200747	88200999	808	906	600	1010	499	833	0.696724727	6.122418492	20.06187096	7.50E-06	0.000321315	+	Exon	AW047730	processed_transcript
3	153624308	153624637	111	117	142	186	106	83	0.697082838	3.464390192	11.40414225	0.000732805	0.009215073	-	Intron	St6galnac3	protein_coding
14	20707696	20707896	531	768	274	667	289	572	0.698338766	5.531606484	12.53571018	0.000399248	0.005951984	+	Exon	Zswim8	protein_coding
14	20707696	20707896	531	768	274	667	289	572	0.698338766	5.531606484	12.53571018	0.000399248	0.005951984	+	Start codon	Zswim8	protein_coding
14	20707696	20707896	531	768	274	667	289	572	0.698338766	5.531606484	12.53571018	0.000399248	0.005951984	+	5'UTR	Zswim8	protein_coding
8	47288047	47288247	207	281	218	361	164	205	0.699264523	4.410648366	20.22258711	6.89E-06	0.00030282	-	5'UTR	Stox2	protein_coding
17	35980495	35980695	3388	5062	2352	5292	2429	3106	0.700553195	8.336478688	27.55004619	1.53E-07	1.59E-05	+	Exon	Gnl1	protein_coding
9	89941507	89941707	108	173	113	190	97	113	0.701042472	3.564162262	16.50877262	4.84E-05	0.001256267	+	Intron	Rasgrf1	protein_coding
8	86884953	86885153	250	362	229	408	162	296	0.701046885	4.65912947	19.62255839	9.43E-06	0.000380629	-	Exon	N4bp1	protein_coding
8	86884953	86885153	250	362	229	408	162	296	0.701046885	4.65912947	19.62255839	9.43E-06	0.000380629	-	Start codon	N4bp1	protein_coding
8	86884953	86885153	250	362	229	408	162	296	0.701046885	4.65912947	19.62255839	9.43E-06	0.000380629	-	5'UTR	N4bp1	protein_coding
4	124657782	124657988	1755	2511	1681	2715	1259	2084	0.701124456	7.487360984	25.44860701	4.54E-07	3.74E-05	+	Exon	Pou3f1	protein_coding
10	9900938	9901142	502	621	392	596	307	614	0.701126251	5.508160602	14.26178098	0.000159062	0.003017599	-	5'UTR	Stxbp5	protein_coding
19	12501406	12501624	285	366	325	403	280	285	0.701758566	4.876371772	16.03859259	6.21E-05	0.001507235	-	5'UTR	Dtx4	protein_coding
15	76656520	76656721	3018	3776	3088	4191	1792	4201	0.704697213	8.224356798	13.51799247	0.000236287	0.00404059	-	Exon	Cyhr1	protein_coding
15	76656520	76656721	3018	3776	3088	4191	1792	4201	0.704697213	8.224356798	13.51799247	0.000236287	0.00404059	-	Start codon	Cyhr1	protein_coding
15	76656520	76656721	3018	3776	3088	4191	1792	4201	0.704697213	8.224356798	13.51799247	0.000236287	0.00404059	-	5'UTR	Cyhr1	protein_coding
14	24004635	24004854	601	557	468	809	340	552	0.706008765	5.620361595	21.54932721	3.45E-06	0.000176361	-	5'UTR	Kcnma1	protein_coding
10	52417656	52417856	291	309	275	386	213	293	0.706799119	4.723082062	22.5659551	2.03E-06	0.000118056	+	Exon	Nus1	protein_coding
10	52417656	52417856	291	309	275	386	213	293	0.706799119	4.723082062	22.5659551	2.03E-06	0.000118056	+	Start codon	Nus1	protein_coding
10	52417656	52417856	291	309	275	386	213	293	0.706799119	4.723082062	22.5659551	2.03E-06	0.000118056	+	5'UTR	Nus1	protein_coding
15	76521919	76522121	2100	1425	1732	2375	1039	1993	0.706882653	7.299638837	17.79578099	2.46E-05	0.000781592	-	5'UTR	Scrt1	protein_coding
16	57121877	57122082	3279	4839	1643	4301	2093	3044	0.707203661	8.171492528	16.70882526	4.36E-05	0.001174482	+	Exon	Tomm70a	protein_coding
19	5424397	5424597	7632	9239	7312	12135	5448	7611	0.707887286	9.521685934	32.83207434	1.00E-08	1.66E-06	-	Exon	Drap1	protein_coding
19	5424397	5424597	7632	9239	7312	12135	5448	7611	0.707887286	9.521685934	32.83207434	1.00E-08	1.66E-06	-	Start codon	Drap1	protein_coding
19	5424397	5424597	7632	9239	7312	12135	5448	7611	0.707887286	9.521685934	32.83207434	1.00E-08	1.66E-06	-	5'UTR	Drap1	protein_coding
15	75746712	75746912	229	241	267	336	137	293	0.708472969	4.470488886	16.26724045	5.50E-05	0.001404876	-	Exon	Mafa	protein_coding
15	75746712	75746912	229	241	267	336	137	293	0.708472969	4.470488886	16.26724045	5.50E-05	0.001404876	-	Stop codon	Mafa	protein_coding
9	21038584	21038818	3506	6626	2049	5756	2455	3473	0.708842946	8.474509429	25.82748386	3.73E-07	3.18E-05	+	Exon	Icam5	protein_coding
16	91044281	91044496	220	260	140	279	125	228	0.709141527	4.220161061	15.632644	7.69E-05	0.001743648	-	Exon	Paxbp1	protein_coding
16	91044281	91044496	220	260	140	279	125	228	0.709141527	4.220161061	15.632644	7.69E-05	0.001743648	-	Start codon	Paxbp1	protein_coding
16	91044281	91044496	220	260	140	279	125	228	0.709141527	4.220161061	15.632644	7.69E-05	0.001743648	-	5'UTR	Paxbp1	protein_coding
19	60226408	60226610	265	542	153	387	214	278	0.710977934	4.789462249	19.22836452	1.16E-05	0.000440892	-	5'UTR	Fam204a	protein_coding
19	60226408	60226610	265	542	153	387	214	278	0.710977934	4.789462249	19.22836452	1.16E-05	0.000440892	+	Exon	4933412A08Rik	pseudogene
4	133498081	133498496	2437	3712	1535	3392	1556	2646	0.711059863	7.838833865	20.39718454	6.29E-06	0.000285048	-	Exon	Trnp1	protein_coding
4	133498081	133498496	2437	3712	1535	3392	1556	2646	0.711059863	7.838833865	20.39718454	6.29E-06	0.000285048	-	Start codon	Trnp1	protein_coding
4	133498081	133498496	2437	3712	1535	3392	1556	2646	0.711059863	7.838833865	20.39718454	6.29E-06	0.000285048	-	5'UTR	Trnp1	protein_coding
11	98837248	98837454	916	1894	592	1565	626	1066	0.713859352	6.629466184	23.56182399	1.21E-06	8.08E-05	+	Exon	Rapgefl1	protein_coding
5	72914769	72914970	580	718	433	783	341	657	0.714006756	5.710116562	18.47154783	1.72E-05	0.00060233	-	Exon	Gm9870	TEC
5	72914769	72914970	580	718	433	783	341	657	0.714006756	5.710116562	18.47154783	1.72E-05	0.00060233	+	Exon	Slain2	protein_coding
5	72914769	72914970	580	718	433	783	341	657	0.714006756	5.710116562	18.47154783	1.72E-05	0.00060233	+	5'UTR	Slain2	protein_coding
8	3451879	3452089	459	643	263	638	336	378	0.714033448	5.350131735	18.40712136	1.78E-05	0.000616506	+	Exon	Arhgef18	protein_coding
7	35120095	35120295	376	453	380	497	287	443	0.714381533	5.194676498	20.63995305	5.54E-06	0.000259641	+	Exon	Cebpa	protein_coding
2	157018194	157018394	116	161	96	197	82	109	0.715588355	3.493741472	17.34928556	3.11E-05	0.000920381	-	Exon	Soga1	protein_coding
2	157018194	157018394	116	161	96	197	82	109	0.715588355	3.493741472	17.34928556	3.11E-05	0.000920381	-	Stop codon	Soga1	protein_coding
2	157018194	157018394	116	161	96	197	82	109	0.715588355	3.493741472	17.34928556	3.11E-05	0.000920381	-	3'UTR	Soga1	protein_coding
12	3309590	3309803	742	1458	464	1051	509	937	0.716723926	6.276652599	16.83968174	4.07E-05	0.001122962	-	5'UTR	Rab10	protein_coding
15	91572942	91573142	171	285	90	238	113	175	0.717925286	4.001096278	16.27239451	5.49E-05	0.001404689	-	Exon	Slc2a13	protein_coding
15	91572942	91573142	171	285	90	238	113	175	0.717925286	4.001096278	16.27239451	5.49E-05	0.001404689	-	Start codon	Slc2a13	protein_coding
15	91572942	91573142	171	285	90	238	113	175	0.717925286	4.001096278	16.27239451	5.49E-05	0.001404689	-	5'UTR	Slc2a13	protein_coding
X	162888151	162888365	504	705	319	693	313	535	0.719130114	5.519032239	19.50875924	1.00E-05	0.000397497	-	Exon	Syap1	protein_coding
X	162888151	162888365	504	705	319	693	313	535	0.719130114	5.519032239	19.50875924	1.00E-05	0.000397497	-	Start codon	Syap1	protein_coding
X	162888151	162888365	504	705	319	693	313	535	0.719130114	5.519032239	19.50875924	1.00E-05	0.000397497	-	5'UTR	Syap1	protein_coding
19	57611131	57611352	243	408	238	292	188	388	0.719563186	4.734822799	9.973419291	0.001588163	0.01634748	+	5'UTR	Atrnl1	protein_coding
9	48662215	48662435	119	144	114	220	68	119	0.719912085	3.520323873	15.14988849	9.93E-05	0.002120397	-	Intron	Zbtb16	protein_coding
16	97170360	97170560	215	309	167	289	142	267	0.721113288	4.378103425	16.62450104	4.56E-05	0.001207244	-	5'UTR	Dscam	protein_coding
13	19619395	19619604	1191	2046	725	1639	895	1256	0.722098707	6.872352985	21.8055871	3.02E-06	0.000160333	-	Exon	Epdr1	protein_coding
13	19619395	19619604	1191	2046	725	1639	895	1256	0.722098707	6.872352985	21.8055871	3.02E-06	0.000160333	-	Start codon	Epdr1	protein_coding
13	19619395	19619604	1191	2046	725	1639	895	1256	0.722098707	6.872352985	21.8055871	3.02E-06	0.000160333	-	5'UTR	Epdr1	protein_coding
2	59214551	59214751	1002	1081	1010	1686	761	832	0.723055382	6.561217501	22.00107591	2.72E-06	0.000149631	+	Exon	Pkp4	protein_coding
2	59214551	59214751	1002	1081	1010	1686	761	832	0.723055382	6.561217501	22.00107591	2.72E-06	0.000149631	+	Start codon	Pkp4	protein_coding
2	59214551	59214751	1002	1081	1010	1686	761	832	0.723055382	6.561217501	22.00107591	2.72E-06	0.000149631	+	5'UTR	Pkp4	protein_coding
15	25984561	25984767	1335	1755	1049	1970	927	1374	0.723168029	6.97626707	29.70159119	5.04E-08	6.39E-06	+	Exon	Zfp622	protein_coding
6	140633555	140633755	210	290	195	329	169	220	0.725861137	4.400358777	23.08852609	1.55E-06	9.68E-05	+	Exon	Aebp2	protein_coding
6	140633555	140633755	210	290	195	329	169	220	0.725861137	4.400358777	23.08852609	1.55E-06	9.68E-05	+	Start codon	Aebp2	protein_coding
6	140633555	140633755	210	290	195	329	169	220	0.725861137	4.400358777	23.08852609	1.55E-06	9.68E-05	+	5'UTR	Aebp2	protein_coding
8	85055734	85055943	355	654	299	629	264	433	0.729722287	5.293849542	24.87035486	6.13E-07	4.73E-05	+	3'UTR	Tnpo2	protein_coding

8	85055734	85055943	355	654	299	629	264	433	0.729722287	5.293849542	24.87035486	6.13E-07	4.73E-05	-	Intron	A230103J11Rik	processed_transcript
15	98806963	98807163	11274	17975	9786	19289	8106	13103	0.730037834	10.21294549	30.76019883	2.92E-08	3.95E-06	-	Exon	Ddn	protein_coding
15	98806963	98807163	11274	17975	9786	19289	8106	13103	0.730037834	10.21294549	30.76019883	2.92E-08	3.95E-06	+	Exon	B130046B21Rik	lincRNA
6	39523725	39523925	64	74	65	74	44	91	0.733701806	2.630477499	10.81545984	0.00100656	0.011704519	-	5'UTR	Dennd2a	protein_coding
19	59345516	59345766	317	363	215	410	257	244	0.734122016	4.769100207	16.54531653	4.75E-05	0.00123553	-	Exon	Pdzd8	protein_coding
19	59345516	59345766	317	363	215	410	257	244	0.734122016	4.769100207	16.54531653	4.75E-05	0.00123553	-	Start codon	Pdzd8	protein_coding
19	59345516	59345766	317	363	215	410	257	244	0.734122016	4.769100207	16.54531653	4.75E-05	0.00123553	-	5'UTR	Pdzd8	protein_coding
19	3261448	3261648	133	269	120	258	98	175	0.73543715	3.964757837	18.79153655	1.46E-05	0.000524078	-	Exon	Ighmbp2	protein_coding
19	3261448	3261648	133	269	120	258	98	175	0.73543715	3.964757837	18.79153655	1.46E-05	0.000524078	-	Stop codon	Ighmbp2	protein_coding
19	3261448	3261648	133	269	120	258	98	175	0.73543715	3.964757837	18.79153655	1.46E-05	0.000524078	-	3'UTR	Ighmbp2	protein_coding
7	127981681	127981881	421	615	441	775	352	436	0.736483031	5.500054442	25.48823993	4.45E-07	3.73E-05	+	Exon	Fus	protein_coding
7	127981681	127981881	421	615	441	775	352	436	0.736483031	5.500054442	25.48823993	4.45E-07	3.73E-05	+	Stop codon	Fus	protein_coding
7	127981681	127981881	421	615	441	775	352	436	0.736483031	5.500054442	25.48823993	4.45E-07	3.73E-05	+	3'UTR	Fus	protein_coding
1	59913197	59913406	490	513	427	651	268	599	0.737835432	5.452957085	16.57737278	4.67E-05	0.001221248	+	Exon	Fam117b	protein_coding
17	31647244	31647455	443	805	198	712	312	365	0.739018265	5.400100082	17.231889	3.31E-05	0.00096176	-	Exon	U2af1	protein_coding
15	78538311	78538511	185	279	131	325	143	152	0.74110651	4.173408961	18.95597649	1.34E-05	0.000491671	-	3'UTR	Sstr3	protein_coding
13	25202098	25202326	407	589	324	545	365	409	0.741154332	5.327568245	20.27673249	6.70E-06	0.00029701	+	Exon	Dcdc2a	protein_coding
11	97500054	97500254	263	422	191	380	215	281	0.741436669	4.723305903	22.5862981	2.01E-06	0.000117504	+	Exon	Arhgap23	protein_coding
11	97500054	97500254	263	422	191	380	215	281	0.741436669	4.723305903	22.5862981	2.01E-06	0.000117504	+	Start codon	Arhgap23	protein_coding
11	97500054	97500254	263	422	191	380	215	281	0.741436669	4.723305903	22.5862981	2.01E-06	0.000117504	+	5'UTR	Arhgap23	protein_coding
11	97500054	97500254	263	422	191	380	215	281	0.741436669	4.723305903	22.5862981	2.01E-06	0.000117504	-	5'UTR	4933428G20Rik	protein_coding
11	97500054	97500254	263	422	191	380	215	281	0.741436669	4.723305903	22.5862981	2.01E-06	0.000117504	+	3'UTR	Arhgap23	protein_coding
14	103346512	103346714	347	509	503	595	277	559	0.741467409	5.379474352	14.46167049	0.000143041	0.002799634	-	Exon	Mycbp2	protein_coding
14	103346512	103346714	347	509	503	595	277	559	0.741467409	5.379474352	14.46167049	0.000143041	0.002799634	-	Start codon	Mycbp2	protein_coding
14	103346512	103346714	347	509	503	595	277	559	0.741467409	5.379474352	14.46167049	0.000143041	0.002799634	-	5'UTR	Mycbp2	protein_coding
11	29373678	29373878	196	302	140	264	117	266	0.741733024	4.26446987	13.53258597	0.000234457	0.004023213	+	5'UTR	Ccdc88a	protein_coding
6	85347942	85348164	924	1423	811	1575	716	1006	0.741877537	6.59338405	33.10783182	8.72E-09	1.51E-06	-	Exon	Rab11fip5	protein_coding
14	54971451	54971651	151	184	66	215	102	96	0.743003693	3.599201089	11.49468236	0.000697956	0.008906591	+	Exon	Mhrt	antisense
14	54971451	54971651	151	184	66	215	102	96	0.743003693	3.599201089	11.49468236	0.000697956	0.008906591	-	Exon	Myh7	protein_coding
8	11008426	11008647	420	486	469	542	270	624	0.743619498	5.39636691	13.36786425	0.000255973	0.004259317	-	Exon	Irs2	protein_coding
8	11008426	11008647	420	486	469	542	270	624	0.743619498	5.39636691	13.36786425	0.000255973	0.004259317	-	Start codon	Irs2	protein_coding
8	11008426	11008647	420	486	469	542	270	624	0.743619498	5.39636691	13.36786425	0.000255973	0.004259317	-	5'UTR	Irs2	protein_coding
10	42502193	42502397	2178	3033	1179	3195	1508	1817	0.744927227	7.598031552	20.37755618	6.36E-06	0.000285048	+	Exon	Snx3	protein_coding
10	42502193	42502397	2178	3033	1179	3195	1508	1817	0.744927227	7.598031552	20.37755618	6.36E-06	0.000285048	+	Start codon	Snx3	protein_coding
10	42502193	42502397	2178	3033	1179	3195	1508	1817	0.744927227	7.598031552	20.37755618	6.36E-06	0.000285048	+	5'UTR	Snx3	protein_coding
2	35979289	35979508	213	224	132	347	147	124	0.746760727	4.131965293	11.49209472	0.000698928	0.008906591	-	Exon	Ttll11	protein_coding
7	44604203	44604403	286	698	210	557	291	284	0.74942558	5.127215121	22.37565393	2.24E-06	0.00012871	+	3'UTR	Kcnc3	protein_coding
5	113795079	113795316	311	499	238	517	256	305	0.749852055	4.993796612	26.55201317	2.57E-07	2.40E-05	-	Exon	Tmem119	protein_coding
6	127769180	127769405	110	163	151	204	81	171	0.753082267	3.704501952	14.8742263	0.000114923	0.002362319	-	5'UTR	Prmt8	protein_coding
4	116463837	116464053	598	585	544	726	416	661	0.753685661	5.733010139	23.46909972	1.27E-06	8.36E-05	-	5'UTR	Mast2	protein_coding
4	116463837	116464053	598	585	544	726	416	661	0.753685661	5.733010139	23.46909972	1.27E-06	8.36E-05	-	Exon	Mast2	protein_coding
4	116463837	116464053	598	585	544	726	416	661	0.753685661	5.733010139	23.46909972	1.27E-06	8.36E-05	-	Start codon	Mast2	protein_coding
1	155558402	155558605	494	909	504	910	404	676	0.756560617	5.866233014	24.42353479	7.73E-07	5.67E-05	+	5'UTR	Acbd6	protein_coding
7	109960193	109960398	1277	1636	911	1699	711	1622	0.758502084	6.878019651	15.23876542	9.47E-05	0.00204008	-	5'UTR	Dennd5a	protein_coding
14	37134949	37135160	453	611	169	690	259	329	0.759293518	5.215461739	11.61898207	0.000652821	0.008523758	-	5'UTR	Ghitm	protein_coding
18	38187544	38187748	689	1466	640	1489	648	748	0.760036035	6.406600507	26.12112482	3.21E-07	2.86E-05	-	3'UTR	Pcdh1	protein_coding
8	70779509	70779709	3806	8005	2181	6130	2659	4596	0.760185142	8.680400745	23.33520105	1.36E-06	8.79E-05	-	Exon	Mast3	protein_coding
8	70779509	70779709	3806	8005	2181	6130	2659	4596	0.760185142	8.680400745	23.33520105	1.36E-06	8.79E-05	-	3'UTR	Mast3	protein_coding
19	46317801	46318005	5462	7400	3615	7847	3531	5880	0.76162491	8.984781247	25.27633605	4.97E-07	4.02E-05	-	Intron	Psd	protein_coding
8	73353205	73353405	352	475	277	518	231	420	0.763006641	5.086927293	22.89135827	1.71E-06	0.000102686	-	5'UTR	Large	protein_coding
6	116338116	116338316	242	307	118	316	139	237	0.764657846	4.342975367	15.59919612	7.83E-05	0.00176666	+	5'UTR	Marchf8	protein_coding
18	37402248	37402486	479	380	634	753	344	474	0.766165295	5.495053689	28.27241889	1.05E-07	1.16E-05	+	Exon	Pcdhb9	protein_coding
3	103279725	103279953	206	197	308	348	141	269	0.766389939	4.431584554	19.48163723	1.02E-05	0.000398386	+	Exon	Trim33	protein_coding
3	103279725	103279953	206	197	308	348	141	269	0.766389939	4.431584554	19.48163723	1.02E-05	0.000398386	+	Start codon	Trim33	protein_coding
3	103279725	103279953	206	197	308	348	141	269	0.766389939	4.431584554	19.48163723	1.02E-05	0.000398386	+	5'UTR	Trim33	protein_coding
11	85234809	85235009	307	439	96	340	184	280	0.766550203	4.63210608	9.597870238	0.001948032	0.018909604	-	5'UTR	Appbp2	protein_coding
11	85234809	85235009	307	439	96	340	184	280	0.766550203	4.63210608	9.597870238	0.001948032	0.018909604	+	Exon	Appbp2os	antisense
18	70568450	70568653	200	311	310	464	143	312	0.767040622	4.672155666	13.04966817	0.000303338	0.0047943	+	Exon	Mbd2	protein_coding
9	43105717	43105927	413	633	519	709	353	605	0.768583201	5.6010588	20.55679597	5.79E-06	0.000268622	-	5'UTR	Arhgef12	protein_coding
16	9994172	9994372	459	729	460	897	381	505	0.770213438	5.67519264	28.75873986	8.20E-08	9.53E-06	-	5'UTR	Grin2a	protein_coding
11	72489615	72489900	423	690	331	680	259	582	0.770284166	5.465800651	17.57084006	2.77E-05	0.000854967	-	Exon	Spns2	protein_coding
11	72489615	72489900	423	690	331	680	259	582	0.770284166	5.465800651	17.57084006	2.77E-05	0.000854967	-	Start codon	Spns2	protein_coding
11	72489615	72489900	423	690	331	680	259	582	0.770284166	5.465800651	17.57084006	2.77E-05	0.000854967	-	5'UTR	Spns2	protein_coding
4	136890426	136890636	1744	2316	1514	2588	1166	2204	0.770547049	7.435646155	24.27612494	8.35E-07	6.02E-05	-	Exon	C1qc	protein_coding
9	120180567	120180771	11412	15392	7813	20475	8164	9164	0.771145405	10.07221595	22.09734532	2.59E-06	0.000143911	+	3'UTR	Mobp	protein_coding
16	32644033	32644244	628	779	643	985	507	703	0.771258431	5.995405565	32.4839694	1.20E-08	1.89E-06	+	Intron	Tnk2	protein_coding
7	120903175	120903375	296	507	181	391	237	338	0.772185908	4.886713545	19.32092709	1.10E-05	0.00042493	+	Exon	Eef2k	protein_coding

16	31297284	31297484	2975	3201	2875	4102	2001	3612	0.772236501	8.139780197	26.5476002	2.57E-07	2.40E-05	-	Exon	Apod	protein_coding
16	31297284	31297484	2975	3201	2875	4102	2001	3612	0.772236501	8.139780197	26.5476002	2.57E-07	2.40E-05	-	Stop codon	Apod	protein_coding
16	31297284	31297484	2975	3201	2875	4102	2001	3612	0.772236501	8.139780197	26.5476002	2.57E-07	2.40E-05	-	3'UTR	Apod	protein_coding
6	87980899	87981122	1156	1755	947	1979	909	1214	0.772576999	6.899911819	36.60172493	1.45E-09	3.98E-07	-	Exon	H1fx	protein_coding
6	87980899	87981122	1156	1755	947	1979	909	1214	0.772576999	6.899911819	36.60172493	1.45E-09	3.98E-07	+	Exon	Gm5577	antisense
15	101232033	101232233	406	750	514	896	429	471	0.773918364	5.697921611	19.87007183	8.29E-06	0.000348623	+	3'UTR	Grasp	protein_coding
17	56605825	56606025	62	157	47	135	52	74	0.774287933	2.970927835	16.46435808	4.96E-05	0.001280547	+	Exon	Safb	protein_coding
1	131097498	131097698	564	666	446	556	297	895	0.774558373	5.697160818	7.789979511	0.005253679	0.037546901	-	Exon	Mapkapk2	protein_coding
1	131097498	131097698	564	666	446	556	297	895	0.774558373	5.697160818	7.789979511	0.005253679	0.037546901	-	Start codon	Mapkapk2	protein_coding
1	131097498	131097698	564	666	446	556	297	895	0.774558373	5.697160818	7.789979511	0.005253679	0.037546901	-	5'UTR	Mapkapk2	protein_coding
7	4690917	4691119	4746	6626	3313	7217	3603	4640	0.777139047	8.830583443	32.99438131	9.24E-09	1.58E-06	+	Exon	Brsk1	protein_coding
7	4690917	4691119	4746	6626	3313	7217	3603	4640	0.777139047	8.830583443	32.99438131	9.24E-09	1.58E-06	+	Start codon	Brsk1	protein_coding
7	4690917	4691119	4746	6626	3313	7217	3603	4640	0.777139047	8.830583443	32.99438131	9.24E-09	1.58E-06	+	5'UTR	Brsk1	protein_coding
6	37299638	37299840	768	988	717	1227	572	876	0.777754588	6.26983723	33.97653609	5.58E-09	1.20E-06	-	Exon	Dgki	protein_coding
10	127059560	127059760	205	274	179	283	133	285	0.777762339	4.348315512	16.79706757	4.16E-05	0.001137365	-	Exon	Marchf9	protein_coding
10	127059560	127059760	205	274	179	283	133	285	0.777762339	4.348315512	16.79706757	4.16E-05	0.001137365	-	Start codon	Marchf9	protein_coding
10	127059560	127059760	205	274	179	283	133	285	0.777762339	4.348315512	16.79706757	4.16E-05	0.001137365	-	5'UTR	Marchf9	protein_coding
5	135868130	135868330	279	357	331	444	220	378	0.778727344	4.910531417	23.03560776	1.59E-06	9.82E-05	+	Stop codon	Srrm3	protein_coding
5	135868130	135868330	279	357	331	444	220	378	0.778727344	4.910531417	23.03560776	1.59E-06	9.82E-05	+	3'UTR	Srrm3	protein_coding
5	135868130	135868330	279	357	331	444	220	378	0.778727344	4.910531417	23.03560776	1.59E-06	9.82E-05	+	Exon	Srrm3	protein_coding
8	25201253	25201455	294	432	303	524	222	373	0.778738599	5.000205972	26.33383314	2.87E-07	2.60E-05	-	Exon	Tacc1	protein_coding
8	25201253	25201455	294	432	303	524	222	373	0.778738599	5.000205972	26.33383314	2.87E-07	2.60E-05	-	Start codon	Tacc1	protein_coding
8	25201253	25201455	294	432	303	524	222	373	0.778738599	5.000205972	26.33383314	2.87E-07	2.60E-05	-	5'UTR	Tacc1	protein_coding
10	82985449	82985649	224	246	193	282	158	266	0.779904719	4.364432952	20.70050669	5.37E-06	0.000255221	+	5'UTR	Chst11	protein_coding
1	156035929	156036129	541	652	420	731	381	606	0.780961553	5.650467532	26.05235012	3.32E-07	2.88E-05	-	Exon	Tor1aip1	protein_coding
1	156035929	156036129	541	652	420	731	381	606	0.780961553	5.650467532	26.05235012	3.32E-07	2.88E-05	+	5'UTR	Tor1aip2	protein_coding
7	5079258	5079486	966	1380	442	1314	638	887	0.782444157	6.405425398	17.84241677	2.40E-05	0.000772231	+	Exon	U2af2	protein_coding
7	5079258	5079486	966	1380	442	1314	638	887	0.782444157	6.405425398	17.84241677	2.40E-05	0.000772231	+	Stop codon	U2af2	protein_coding
7	5079258	5079486	966	1380	442	1314	638	887	0.782444157	6.405425398	17.84241677	2.40E-05	0.000772231	+	3'UTR	U2af2	protein_coding
11	59662577	59662858	947	1340	734	1453	700	1067	0.786281244	6.554705374	32.73223955	1.06E-08	1.69E-06	+	Exon	Mprip	protein_coding
11	59662577	59662858	947	1340	734	1453	700	1067	0.786281244	6.554705374	32.73223955	1.06E-08	1.69E-06	+	Start codon	Mprip	protein_coding
11	59662577	59662858	947	1340	734	1453	700	1067	0.786281244	6.554705374	32.73223955	1.06E-08	1.69E-06	+	5'UTR	Mprip	protein_coding
1	42697071	42697271	150	90	101	181	71	123	0.788218159	3.399591492	15.40113544	8.69E-05	0.001905268	+	Exon	Pou3f3	protein_coding
1	42697071	42697271	150	90	101	181	71	123	0.788218159	3.399591492	15.40113544	8.69E-05	0.001905268	+	Start codon	Pou3f3	protein_coding
1	42697071	42697271	150	90	101	181	71	123	0.788218159	3.399591492	15.40113544	8.69E-05	0.001905268	+	5'UTR	Pou3f3	protein_coding
8	79639546	79639746	97	152	88	179	64	125	0.78884281	3.386204046	18.76672849	1.48E-05	0.000529016	+	5'UTR	Otud4	protein_coding
15	89557707	89557982	1082	2275	614	1828	808	1249	0.790196287	6.881255768	28.77466702	8.13E-08	9.53E-06	+	Exon	Shank3	protein_coding
15	89557707	89557982	1082	2275	614	1828	808	1249	0.790196287	6.881255768	28.77466702	8.13E-08	9.53E-06	+	Stop codon	Shank3	protein_coding
15	89557707	89557982	1082	2275	614	1828	808	1249	0.790196287	6.881255768	28.77466702	8.13E-08	9.53E-06	+	3'UTR	Shank3	protein_coding
7	73558092	73558292	823	1165	399	1107	444	936	0.792280988	6.188898988	14.28845521	0.000156824	0.003000115	-	Exon	1810026B05Rik	processed_transcript
2	157470593	157470801	710	1371	552	1414	616	741	0.796019384	6.338327298	33.72314218	6.35E-09	1.27E-06	+	3'UTR	Src	protein_coding
1	89580628	89580828	173	236	225	316	170	205	0.796093099	4.310701602	22.74152844	1.85E-06	0.00010903	+	5'UTR	Agap1	protein_coding
1	66468237	66468437	803	978	645	1252	477	972	0.800174945	6.254735477	23.23066087	1.44E-06	9.20E-05	+	5'UTR	Unc80	protein_coding
14	31251522	31251722	466	1237	353	961	457	578	0.800609738	5.929329813	34.15497948	5.09E-09	1.12E-06	+	Exon	Bap1	protein_coding
14	31251522	31251722	466	1237	353	961	457	578	0.800609738	5.929329813	34.15497948	5.09E-09	1.12E-06	+	Start codon	Bap1	protein_coding
14	31251522	31251722	466	1237	353	961	457	578	0.800609738	5.929329813	34.15497948	5.09E-09	1.12E-06	+	5'UTR	Bap1	protein_coding
7	16455154	16455376	374	565	255	591	321	346	0.800819975	5.211463552	26.40722475	2.77E-07	2.53E-05	+	Exon	Tmem160	protein_coding
7	16455154	16455376	374	565	255	591	321	346	0.800819975	5.211463552	26.40722475	2.77E-07	2.53E-05	+	3'UTR	Tmem160	protein_coding
18	21299783	21299999	725	860	519	1118	435	793	0.805203884	6.051034952	25.41848082	4.61E-07	3.77E-05	-	5'UTR	Garem	protein_coding
7	15945878	15946078	685	978	398	1111	490	616	0.805543262	6.002195531	25.99924288	3.42E-07	2.94E-05	-	Exon	Gltscr2	protein_coding
7	15945878	15946078	685	978	398	1111	490	616	0.805543262	6.002195531	25.99924288	3.42E-07	2.94E-05	-	Start codon	Gltscr2	protein_coding
7	15945878	15946078	685	978	398	1111	490	616	0.805543262	6.002195531	25.99924288	3.42E-07	2.94E-05	-	5'UTR	Gltscr2	protein_coding
1	39576897	39577317	559	1081	476	842	474	843	0.806171965	6.023952101	19.70393246	9.04E-06	0.000367763	-	Exon	Rnf149	protein_coding
1	39576897	39577317	559	1081	476	842	474	843	0.806171965	6.023952101	19.70393246	9.04E-06	0.000367763	-	Start codon	Rnf149	protein_coding
1	39576897	39577317	559	1081	476	842	474	843	0.806171965	6.023952101	19.70393246	9.04E-06	0.000367763	-	5'UTR	Rnf149	protein_coding
2	18677074	18677277	277	347	178	394	199	273	0.806514579	4.648677577	25.13276231	5.35E-07	4.30E-05	+	5'UTR	Bmi1	protein_coding
19	36834299	36834607	684	1126	420	984	381	967	0.806880641	6.095353487	13.72366616	0.000211769	0.003711216	+	Exon	Tnks2	protein_coding
19	36834299	36834607	684	1126	420	984	381	967	0.806880641	6.095353487	13.72366616	0.000211769	0.003711216	+	Start codon	Tnks2	protein_coding
19	36834299	36834607	684	1126	420	984	381	967	0.806880641	6.095353487	13.72366616	0.000211769	0.003711216	+	5'UTR	Tnks2	protein_coding
19	10525570	10525770	127	251	49	165	103	130	0.812215861	3.64117738	14.85319902	0.000116211	0.002373197	+	Intron	Cpsf7	protein_coding
17	56563154	56563361	242	352	152	370	170	260	0.81248178	4.534976036	25.45414083	4.53E-07	3.74E-05	-	Stop codon	Safb2	protein_coding
17	56563154	56563361	242	352	152	370	170	260	0.81248178	4.534976036	25.45414083	4.53E-07	3.74E-05	-	3'UTR	Safb2	protein_coding
17	56563154	56563361	242	352	152	370	170	260	0.81248178	4.534976036	25.45414083	4.53E-07	3.74E-05	-	Exon	Safb2	protein_coding
8	84706656	84706857	2700	4725	1991	4631	2196	3129	0.814405807	8.19336327	40.53537711	1.93E-10	6.36E-08	-	3'UTR	Nfix	protein_coding
16	90386204	90386423	496	497	534	770	352	579	0.814530156	5.590021897	33.42366192	7.41E-09	1.41E-06	+	5'UTR	Hunk	protein_coding
3	104219965	104220165	249	281	280	413	179	315	0.814841927	4.675380212	27.29158103	1.75E-07	1.76E-05	-	Exon	Magi3	protein_coding
3	104219965	104220165	249	281	280	413	179	315	0.814841927	4.675380212	27.29158103	1.75E-07	1.76E-05	-	Start codon	Magi3	protein_coding

3	104219965	104220165	249	281	280	413	179	315	0.814841927	4.675380212	27.29158103	1.75E-07	1.76E-05	-	5'UTR	Magi3	protein_coding
18	38188044	38188378	457	785	361	794	382	536	0.815894346	5.642663721	35.55233099	2.48E-09	6.14E-07	-	3'UTR	Pcdh1	protein_coding
2	18997988	18998205	591	601	377	784	410	533	0.818283583	5.636595237	24.9294908	5.95E-07	4.63E-05	-	Exon	Pip4k2a	protein_coding
2	18997988	18998205	591	601	377	784	410	533	0.818283583	5.636595237	24.9294908	5.95E-07	4.63E-05	-	Start codon	Pip4k2a	protein_coding
2	18997988	18998205	591	601	377	784	410	533	0.818283583	5.636595237	24.9294908	5.95E-07	4.63E-05	-	5'UTR	Pip4k2a	protein_coding
19	41263849	41264049	726	903	488	928	459	903	0.8183998	6.058973257	19.2209135	1.16E-05	0.00044092	-	Exon	Tm9sf3	protein_coding
19	41263849	41264049	726	903	488	928	459	903	0.8183998	6.058973257	19.2209135	1.16E-05	0.00044092	-	Start codon	Tm9sf3	protein_coding
19	41263849	41264049	726	903	488	928	459	903	0.8183998	6.058973257	19.2209135	1.16E-05	0.00044092	-	5'UTR	Tm9sf3	protein_coding
11	97501100	97501300	493	781	426	996	454	436	0.818670194	5.747199884	23.70343524	1.12E-06	7.66E-05	+	Exon	Arhgap23	protein_coding
11	97501100	97501300	493	781	426	996	454	436	0.818670194	5.747199884	23.70343524	1.12E-06	7.66E-05	+	Stop codon	Arhgap23	protein_coding
11	97501100	97501300	493	781	426	996	454	436	0.818670194	5.747199884	23.70343524	1.12E-06	7.66E-05	+	3'UTR	Arhgap23	protein_coding
1	191396789	191396999	292	421	189	441	231	294	0.8201613	4.817126871	27.5798033	1.51E-07	1.59E-05	-	5'UTR	Ppp2r5a	protein_coding
17	34898706	34899228	544	1169	585	1029	510	894	0.822687871	6.162834251	21.93865766	2.82E-06	0.000153724	+	Exon	Ehmt2	protein_coding
17	34898706	34899228	544	1169	585	1029	510	894	0.822687871	6.162834251	21.93865766	2.82E-06	0.000153724	+	Start codon	Ehmt2	protein_coding
17	34898706	34899228	544	1169	585	1029	510	894	0.822687871	6.162834251	21.93865766	2.82E-06	0.000153724	+	5'UTR	Ehmt2	protein_coding
10	7955944	7956144	179	216	225	305	125	270	0.826748321	4.29511034	20.06985235	7.47E-06	0.000321315	-	5'UTR	Tab2	protein_coding
1	75479097	75479297	179	472	190	288	214	322	0.827057916	4.674978219	13.77354684	0.00020622	0.003633288	-	Exon	Chpf	protein_coding
1	75479097	75479297	179	472	190	288	214	322	0.827057916	4.674978219	13.77354684	0.00020622	0.003633288	-	Start codon	Chpf	protein_coding
1	75479097	75479297	179	472	190	288	214	322	0.827057916	4.674978219	13.77354684	0.00020622	0.003633288	-	5'UTR	Chpf	protein_coding
13	95222766	95222979	374	439	294	481	214	532	0.827690158	5.133674039	15.11978361	0.0001009	0.002136808	-	Exon	Pde8b	protein_coding
13	95222766	95222979	374	439	294	481	214	532	0.827690158	5.133674039	15.11978361	0.0001009	0.002136808	-	Start codon	Pde8b	protein_coding
13	95222766	95222979	374	439	294	481	214	532	0.827690158	5.133674039	15.11978361	0.0001009	0.002136808	-	5'UTR	Pde8b	protein_coding
18	37302059	37302259	306	125	391	309	160	339	0.828102638	4.579975528	21.80704088	3.01E-06	0.000160333	+	Exon	Pcdhb3	protein_coding
8	40308121	40308334	509	1243	321	753	441	759	0.828878164	5.938997306	18.71575703	1.52E-05	0.000537504	-	Exon	Fgf20	protein_coding
8	40308121	40308334	509	1243	321	753	441	759	0.828878164	5.938997306	18.71575703	1.52E-05	0.000537504	-	Start codon	Fgf20	protein_coding
8	40308121	40308334	509	1243	321	753	441	759	0.828878164	5.938997306	18.71575703	1.52E-05	0.000537504	-	5'UTR	Fgf20	protein_coding
8	40308121	40308334	509	1243	321	753	441	759	0.828878164	5.938997306	18.71575703	1.52E-05	0.000537504	+	Exon	Micu3	protein_coding
1	86046053	86046253	131	266	121	224	95	233	0.83266886	4.005951019	14.91516668	0.000112455	0.00232533	+	Exon	2810459M11Rik	protein_coding
8	119446931	119447131	264	423	214	476	177	352	0.835385162	4.828383648	25.0700993	5.53E-07	4.37E-05	+	Exon	Necab2	protein_coding
8	119446931	119447131	264	423	214	476	177	352	0.835385162	4.828383648	25.0700993	5.53E-07	4.37E-05	+	Start codon	Necab2	protein_coding
8	119446931	119447131	264	423	214	476	177	352	0.835385162	4.828383648	25.0700993	5.53E-07	4.37E-05	+	5'UTR	Necab2	protein_coding
12	59218969	59219169	297	557	171	320	251	419	0.839789786	4.955585541	12.21856212	0.000473164	0.00670983	-	Exon	Fbxo33	protein_coding
16	35769102	35769321	626	981	326	867	363	812	0.839883987	5.902674539	15.65709192	7.59E-05	0.001725208	-	Exon	Dirc2	protein_coding
16	35769102	35769321	626	981	326	867	363	812	0.839883987	5.902674539	15.65709192	7.59E-05	0.001725208	-	Start codon	Dirc2	protein_coding
16	35769102	35769321	626	981	326	867	363	812	0.839883987	5.902674539	15.65709192	7.59E-05	0.001725208	-	5'UTR	Dirc2	protein_coding
17	86963417	86963629	354	445	293	561	245	429	0.841529015	5.124455453	30.39073169	3.53E-08	4.65E-06	+	Exon	Rhoq	protein_coding
17	86963417	86963629	354	445	293	561	245	429	0.841529015	5.124455453	30.39073169	3.53E-08	4.65E-06	+	Start codon	Rhoq	protein_coding
17	86963417	86963629	354	445	293	561	245	429	0.841529015	5.124455453	30.39073169	3.53E-08	4.65E-06	+	5'UTR	Rhoq	protein_coding
4	129984839	129985041	479	892	287	668	277	763	0.841563024	5.664753078	11.99479829	0.000533493	0.007293278	+	5'UTR	Adgrb2	protein_coding
11	100188561	100188761	29	69	56	112	40	30	0.84239412	2.319523228	8.45802352	0.003634366	0.029145035	-	Exon	Krt9	protein_coding
15	38078496	38078705	478	548	330	562	319	603	0.843544482	5.433484811	18.50673621	1.69E-05	0.000595519	-	5'UTR	Ubr5	protein_coding
5	30588173	30588373	221	277	257	312	218	295	0.844608841	4.589524258	22.19908104	2.46E-06	0.000139621	-	Exon	Gm9899	processed_transcript
5	30588173	30588373	221	277	257	312	218	295	0.844608841	4.589524258	22.19908104	2.46E-06	0.000139621	+	Exon	Kcnk3	protein_coding
5	30588173	30588373	221	277	257	312	218	295	0.844608841	4.589524258	22.19908104	2.46E-06	0.000139621	+	Start codon	Kcnk3	protein_coding
5	30588173	30588373	221	277	257	312	218	295	0.844608841	4.589524258	22.19908104	2.46E-06	0.000139621	+	5'UTR	Kcnk3	protein_coding
X	7921949	7922212	8743	15572	5037	14695	6845	9377	0.8467039	9.832466344	35.15158715	3.05E-09	7.01E-07	+	Exon	Pcsk1n	protein_coding
18	37670842	37671067	392	345	431	550	294	462	0.851236621	5.216167383	33.84025181	5.98E-09	1.26E-06	+	Exon	Pcdhga2	protein_coding
4	151145373	151145599	130	308	88	230	124	173	0.851510614	3.991599975	25.11253472	5.41E-07	4.31E-05	-	Exon	Camta1	protein_coding
14	54912562	54912829	3323	4448	1414	4146	2135	3434	0.851593457	8.168797333	16.24619504	5.56E-05	0.001410023	-	5'UTR	Slc22a17	protein_coding
10	63023391	63023591	113	161	65	118	64	177	0.85313096	3.401858194	9.509384893	0.002044237	0.019578717	-	Intron	Hnrnph3	protein_coding
19	6954130	6954330	184	195	103	222	119	195	0.853335394	3.941152177	18.96675844	1.33E-05	0.000491671	-	Exon	Plcb3	protein_coding
15	12321469	12321889	1114	1658	736	1838	750	1324	0.853452652	6.800391201	30.16894309	3.96E-08	5.15E-06	-	Exon	1810049J17Rik	protein_coding
15	12321469	12321889	1114	1658	736	1838	750	1324	0.853452652	6.800391201	30.16894309	3.96E-08	5.15E-06	+	Exon	Golph3	protein_coding
15	12321469	12321889	1114	1658	736	1838	750	1324	0.853452652	6.800391201	30.16894309	3.96E-08	5.15E-06	+	Start codon	Golph3	protein_coding
15	12321469	12321889	1114	1658	736	1838	750	1324	0.853452652	6.800391201	30.16894309	3.96E-08	5.15E-06	+	5'UTR	Golph3	protein_coding
7	44604423	44604651	410	694	389	744	389	518	0.853947864	5.574777805	35.89213811	2.09E-09	5.42E-07	+	3'UTR	Kcnc3	protein_coding
1	75142805	75143009	1056	1654	571	1464	732	1272	0.853958368	6.679749874	21.77820235	3.06E-06	0.000161769	+	Exon	Fam134a	protein_coding
1	75142805	75143009	1056	1654	571	1464	732	1272	0.853958368	6.679749874	21.77820235	3.06E-06	0.000161769	+	Start codon	Fam134a	protein_coding
1	75142805	75143009	1056	1654	571	1464	732	1272	0.853958368	6.679749874	21.77820235	3.06E-06	0.000161769	+	5'UTR	Fam134a	protein_coding
8	111537216	111537439	507	919	375	843	401	696	0.858775779	5.823237695	29.66279827	5.14E-08	6.43E-06	+	Exon	Znrf1	protein_coding
9	57765368	57765568	104	63	88	146	37	115	0.861991513	3.020220485	13.52357986	0.000235585	0.00403556	-	Exon	Clk3	protein_coding
9	57765368	57765568	104	63	88	146	37	115	0.861991513	3.020220485	13.52357986	0.000235585	0.00403556	-	Start codon	Clk3	protein_coding
9	57765368	57765568	104	63	88	146	37	115	0.861991513	3.020220485	13.52357986	0.000235585	0.00403556	-	5'UTR	Clk3	protein_coding
15	82147242	82147442	675	1047	440	1122	585	664	0.865281002	6.105109787	33.7023074	6.42E-09	1.27E-06	+	Exon	Srebf2	protein_coding
15	82147242	82147442	675	1047	440	1122	585	664	0.865281002	6.105109787	33.7023074	6.42E-09	1.27E-06	+	Start codon	Srebf2	protein_coding
15	82147242	82147442	675	1047	440	1122	585	664	0.865281002	6.105109787	33.7023074	6.42E-09	1.27E-06	+	5'UTR	Srebf2	protein_coding
6	38504444	38504665	28	20	52	45	21	39	0.865682898	1.627869145	11.49120486	0.000699263	0.008906591	+	3'UTR	Ubn2	protein_coding

4	129120887	129121106	298	718	180	615	191	433	0.865696129	5.17507283	20.70025328	5.37E-06	0.000255221	-	5'UTR	Hpca	protein_coding
13	75707501	75707701	196	163	101	236	101	195	0.865942064	3.890846153	16.12737175	5.92E-05	0.001470727	+	Exon	Ell2	protein_coding
13	75707501	75707701	196	163	101	236	101	195	0.865942064	3.890846153	16.12737175	5.92E-05	0.001470727	+	Start codon	Ell2	protein_coding
13	75707501	75707701	196	163	101	236	101	195	0.865942064	3.890846153	16.12737175	5.92E-05	0.001470727	+	5'UTR	Ell2	protein_coding
1	52232705	52232921	2096	3771	1131	3358	1492	2536	0.866667254	7.764152138	27.1534651	1.88E-07	1.88E-05	-	Exon	Gls	protein_coding
10	17947719	17947936	519	590	568	821	404	703	0.871354493	5.764811825	32.7545761	1.05E-08	1.69E-06	-	Exon	Heca	protein_coding
10	17947719	17947936	519	590	568	821	404	703	0.871354493	5.764811825	32.7545761	1.05E-08	1.69E-06	-	Start codon	Heca	protein_coding
10	17947719	17947936	519	590	568	821	404	703	0.871354493	5.764811825	32.7545761	1.05E-08	1.69E-06	-	5'UTR	Heca	protein_coding
17	57059611	57059813	2142	4090	1251	3737	1589	2637	0.871651748	7.863609279	32.88613791	9.77E-09	1.64E-06	-	Exon	Slc25a23	protein_coding
17	57059611	57059813	2142	4090	1251	3737	1589	2637	0.871651748	7.863609279	32.88613791	9.77E-09	1.64E-06	-	Start codon	Slc25a23	protein_coding
17	57059611	57059813	2142	4090	1251	3737	1589	2637	0.871651748	7.863609279	32.88613791	9.77E-09	1.64E-06	-	5'UTR	Slc25a23	protein_coding
17	57059611	57059813	2142	4090	1251	3737	1589	2637	0.871651748	7.863609279	32.88613791	9.77E-09	1.64E-06	+	Intron	Crb3	protein_coding
2	28205722	28205922	14763	27906	9794	23235	9733	23050	0.871677872	10.68066689	17.91589951	2.31E-05	0.000753504	+	5'UTR	Olfm1	protein_coding
1	23382912	23383112	1296	2183	911	2156	922	1795	0.879670035	7.129038347	27.85069241	1.31E-07	1.42E-05	-	Exon	Ogfrl1	protein_coding
1	23382912	23383112	1296	2183	911	2156	922	1795	0.879670035	7.129038347	27.85069241	1.31E-07	1.42E-05	-	Start codon	Ogfrl1	protein_coding
1	23382912	23383112	1296	2183	911	2156	922	1795	0.879670035	7.129038347	27.85069241	1.31E-07	1.42E-05	-	5'UTR	Ogfrl1	protein_coding
11	83412384	83412593	3117	4448	2249	4687	2274	4078	0.882160446	8.310024793	29.94887441	4.44E-08	5.69E-06	+	5'UTR	Rasl10b	protein_coding
2	24762969	24763169	135	246	167	307	125	208	0.883994916	4.149918193	25.58937344	4.22E-07	3.57E-05	-	Exon	Cacna1b	protein_coding
2	24762969	24763169	135	246	167	307	125	208	0.883994916	4.149918193	25.58937344	4.22E-07	3.57E-05	-	Start codon	Cacna1b	protein_coding
2	24762969	24763169	135	246	167	307	125	208	0.883994916	4.149918193	25.58937344	4.22E-07	3.57E-05	-	5'UTR	Cacna1b	protein_coding
4	155222470	155222693	1580	1898	1045	2235	985	1984	0.885030043	7.201317226	23.70587177	1.12E-06	7.66E-05	-	Exon	Ski	protein_coding
4	155222470	155222693	1580	1898	1045	2235	985	1984	0.885030043	7.201317226	23.70587177	1.12E-06	7.66E-05	-	Start codon	Ski	protein_coding
4	155222470	155222693	1580	1898	1045	2235	985	1984	0.885030043	7.201317226	23.70587177	1.12E-06	7.66E-05	-	5'UTR	Ski	protein_coding
4	124850298	124850502	576	823	396	857	516	617	0.88859584	5.858019199	32.17078982	1.41E-08	2.15E-06	-	Exon	1110065P20Rik	protein_coding
4	124850298	124850502	576	823	396	857	516	617	0.88859584	5.858019199	32.17078982	1.41E-08	2.15E-06	-	5'UTR	1110065P20Rik	protein_coding
7	25282553	25282753	68	171	83	134	51	171	0.890465747	3.350494074	9.888196822	0.001663423	0.016880156	+	5'UTR	Cic	protein_coding
4	56990140	56990340	632	1107	476	1130	449	932	0.898842611	6.153449288	27.05285892	1.98E-07	1.96E-05	-	Exon	Frrs1l	protein_coding
4	56990140	56990340	632	1107	476	1130	449	932	0.898842611	6.153449288	27.05285892	1.98E-07	1.96E-05	-	Start codon	Frrs1l	protein_coding
4	56990140	56990340	632	1107	476	1130	449	932	0.898842611	6.153449288	27.05285892	1.98E-07	1.96E-05	-	5'UTR	Frrs1l	protein_coding
17	39844951	39845256	11271	22761	6097	18846	8181	15173	0.899730457	10.28359744	26.66470749	2.42E-07	2.32E-05	+	Exon	Gm26917	lincRNA
7	90130364	90130604	1083	1485	477	1636	763	1004	0.900245775	6.609667188	22.17880075	2.48E-06	0.000140297	+	Exon	Picalm	protein_coding
7	90130364	90130604	1083	1485	477	1636	763	1004	0.900245775	6.609667188	22.17880075	2.48E-06	0.000140297	+	Start codon	Picalm	protein_coding
7	90130364	90130604	1083	1485	477	1636	763	1004	0.900245775	6.609667188	22.17880075	2.48E-06	0.000140297	+	5'UTR	Picalm	protein_coding
11	57801723	57801967	1067	1437	758	1541	716	1487	0.902317923	6.734065967	23.61334928	1.18E-06	7.97E-05	+	5'UTR	Sap30l	protein_coding
4	46471240	46471440	125	126	97	199	84	133	0.904153006	3.511168302	27.01696903	2.02E-07	1.97E-05	+	Exon	Anp32b	protein_coding
17	39848215	39848477	1436	3498	771	2464	1140	2080	0.90522992	7.431516211	26.42263225	2.74E-07	2.53E-05	-	Exon	Rn18s-rs5	rRNA
17	39848215	39848477	1436	3498	771	2464	1140	2080	0.90522992	7.431516211	26.42263225	2.74E-07	2.53E-05	+	Exon	Gm42418	lincRNA
14	39472682	39472882	319	486	273	434	230	554	0.90672458	5.130195298	15.72315284	7.33E-05	0.00168697	-	Exon	Nrg3	protein_coding
14	39472682	39472882	319	486	273	434	230	554	0.90672458	5.130195298	15.72315284	7.33E-05	0.00168697	-	Start codon	Nrg3	protein_coding
14	39472682	39472882	319	486	273	434	230	554	0.90672458	5.130195298	15.72315284	7.33E-05	0.00168697	-	5'UTR	Nrg3	protein_coding
9	43222320	43222520	96	108	139	233	69	120	0.907146863	3.486525716	19.49781965	1.01E-05	0.000397497	-	3'UTR	Oaf	protein_coding
9	62811400	62811600	273	556	204	403	249	445	0.913815367	5.030091627	20.47368541	6.05E-06	0.000279209	-	5'UTR	Fem1b	protein_coding
15	9071470	9071670	411	583	224	622	282	482	0.914492956	5.299409195	26.65117184	2.44E-07	2.32E-05	+	Exon	Nadk2	protein_coding
15	9071470	9071670	411	583	224	622	282	482	0.914492956	5.299409195	26.65117184	2.44E-07	2.32E-05	+	Start codon	Nadk2	protein_coding
15	9071470	9071670	411	583	224	622	282	482	0.914492956	5.299409195	26.65117184	2.44E-07	2.32E-05	+	5'UTR	Nadk2	protein_coding
19	43674543	43674754	740	1015	358	933	620	744	0.916049829	6.090258132	20.6595233	5.49E-06	0.00025946	-	Exon	Slc25a28	protein_coding
7	82867371	82867571	59	75	88	111	51	103	0.924221152	2.866967246	19.85940574	8.34E-06	0.000348954	+	5'UTR	Mex3b	protein_coding
7	123982789	123982989	119	261	87	219	112	178	0.930505084	3.885589299	28.27559429	1.05E-07	1.16E-05	+	5'UTR	Hs3st4	protein_coding
7	123982789	123982989	119	261	87	219	112	178	0.930505084	3.885589299	28.27559429	1.05E-07	1.16E-05	-	Intron	Gm27040	antisense
17	24720306	24720506	3499	7917	1779	6526	2728	4580	0.934190809	8.673496142	33.77258522	6.19E-09	1.27E-06	+	Exon	Rps2	protein_coding
4	43443305	43443532	1135	1630	762	1484	806	1722	0.935521628	6.85612126	19.25053007	1.15E-05	0.000437485	+	Exon	Tesk1	protein_coding
4	43443305	43443532	1135	1630	762	1484	806	1722	0.935521628	6.85612126	19.25053007	1.15E-05	0.000437485	+	Start codon	Tesk1	protein_coding
4	43443305	43443532	1135	1630	762	1484	806	1722	0.935521628	6.85612126	19.25053007	1.15E-05	0.000437485	+	5'UTR	Tesk1	protein_coding
6	134830194	134830394	450	486	408	719	377	519	0.93825935	5.48966877	45.40562997	1.60E-11	7.54E-09	+	5'UTR	Crebl2	protein_coding
7	44312637	44312837	360	698	342	797	387	452	0.93854626	5.525408949	40.68262203	1.79E-10	6.10E-08	+	Exon	Shank1	protein_coding
7	44312637	44312837	360	698	342	797	387	452	0.93854626	5.525408949	40.68262203	1.79E-10	6.10E-08	+	Start codon	Shank1	protein_coding
7	44312637	44312837	360	698	342	797	387	452	0.93854626	5.525408949	40.68262203	1.79E-10	6.10E-08	+	5'UTR	Shank1	protein_coding
7	6132434	6132648	130	201	95	287	92	143	0.943298975	3.813002762	27.46118231	1.60E-07	1.64E-05	-	Exon	Zfp787	protein_coding
11	30771885	30772085	511	476	222	463	360	547	0.948902725	5.324796572	12.91396164	0.00032614	0.005092528	+	Exon	Psme4	protein_coding
11	30771885	30772085	511	476	222	463	360	547	0.948902725	5.324796572	12.91396164	0.00032614	0.005092528	+	Start codon	Psme4	protein_coding
11	30771885	30772085	511	476	222	463	360	547	0.948902725	5.324796572	12.91396164	0.00032614	0.005092528	+	5'UTR	Psme4	protein_coding
19	5843826	5844124	2493	1707	1986	3557	1372	2723	0.950109119	7.692356461	29.3375912	6.08E-08	7.42E-06	-	Exon	Neat1	lincRNA
11	69556082	69556293	921	1636	550	1778	796	998	0.952055803	6.661552433	43.80278958	3.63E-11	1.44E-08	-	Exon	Efnb3	protein_coding
10	116473531	116473772	537	602	271	596	333	709	0.955586665	5.547990782	15.40330064	8.68E-05	0.001905268	+	Exon	Kcnmb4os2	antisense
10	116473531	116473772	537	602	271	596	333	709	0.955586665	5.547990782	15.40330064	8.68E-05	0.001905268	-	5'UTR	Kcnmb4	protein_coding
8	104395569	104395771	404	571	249	523	363	503	0.962944017	5.33875871	24.99856986	5.74E-07	4.50E-05	-	Exon	Cmtm4	protein_coding
8	104395569	104395771	404	571	249	523	363	503	0.962944017	5.33875871	24.99856986	5.74E-07	4.50E-05	-	Start codon	Cmtm4	protein_coding

8	104395569	104395771	404	571	249	523	363	503	0.962944017	5.33875871	24.99856986	5.74E-07	4.50E-05	-	5'UTR	Cmtm4	protein_coding
19	6920347	6920547	97	106	37	119	48	119	0.964136102	2.986229878	13.55079069	0.000232194	0.00399826	-	Exon	Esrra	protein_coding
19	6920347	6920547	97	106	37	119	48	119	0.964136102	2.986229878	13.55079069	0.000232194	0.00399826	-	Start codon	Esrra	protein_coding
19	6920347	6920547	97	106	37	119	48	119	0.964136102	2.986229878	13.55079069	0.000232194	0.00399826	-	5'UTR	Esrra	protein_coding
3	88058306	88058506	429	1100	277	928	384	642	0.970261939	5.827188906	42.20086367	8.24E-11	2.91E-08	-	Exon	Apoa1bp	protein_coding
3	88058306	88058506	429	1100	277	928	384	642	0.970261939	5.827188906	42.20086367	8.24E-11	2.91E-08	-	Start codon	Apoa1bp	protein_coding
3	88058306	88058506	429	1100	277	928	384	642	0.970261939	5.827188906	42.20086367	8.24E-11	2.91E-08	-	5'UTR	Apoa1bp	protein_coding
2	104494148	104494348	238	263	168	317	172	322	0.97603321	4.495720142	26.07401119	3.29E-07	2.88E-05	-	5'UTR	Hipk3	protein_coding
10	82763564	82763764	243	327	161	345	174	356	0.980819873	4.613185297	24.10884053	9.10E-07	6.38E-05	-	5'UTR	Nfyb	protein_coding
7	25282237	25282437	156	213	88	208	108	224	0.985821242	3.923395508	20.38665681	6.33E-06	0.000285048	+	5'UTR	Cic	protein_coding
19	5845176	5845382	904	1204	814	1836	600	1328	0.987659351	6.644631084	30.56137203	3.23E-08	4.32E-06	-	Exon	Neat1	lincRNA
5	101765214	101765418	856	1057	439	1306	632	935	1.001844118	6.319231117	30.90904591	2.70E-08	3.87E-06	+	Exon	Cds1	protein_coding
5	101765214	101765418	856	1057	439	1306	632	935	1.001844118	6.319231117	30.90904591	2.70E-08	3.87E-06	+	Start codon	Cds1	protein_coding
5	101765214	101765418	856	1057	439	1306	632	935	1.001844118	6.319231117	30.90904591	2.70E-08	3.87E-06	+	5'UTR	Cds1	protein_coding
8	36249087	36249313	216	180	138	272	151	243	1.003083498	4.189951416	27.77457204	1.36E-07	1.46E-05	-	Exon	Lonrf1	protein_coding
13	117602727	117602942	555	634	338	914	366	661	1.006057664	5.711621171	33.12815327	8.63E-09	1.51E-06	+	Exon	Hcn1	protein_coding
2	122702492	122702694	104	274	83	202	124	180	1.00836862	3.887299421	28.32990046	1.02E-07	1.15E-05	-	5'UTR	Slc30a4	protein_coding
19	5844352	5844552	730	863	604	1264	544	1008	1.016251081	6.250498561	43.18349197	4.98E-11	1.89E-08	-	Exon	Neat1	lincRNA
19	5844352	5844552	730	863	604	1264	544	1008	1.016251081	6.250498561	43.18349197	4.98E-11	1.89E-08	-	Exon	Gm27533	misc_RNA
13	68999270	68999470	456	561	310	618	297	754	1.039532136	5.522178417	19.67447539	9.18E-06	0.000371945	-	Exon	Adcy2	protein_coding
13	68999270	68999470	456	561	310	618	297	754	1.039532136	5.522178417	19.67447539	9.18E-06	0.000371945	-	Start codon	Adcy2	protein_coding
13	68999270	68999470	456	561	310	618	297	754	1.039532136	5.522178417	19.67447539	9.18E-06	0.000371945	-	5'UTR	Adcy2	protein_coding
7	31116913	31117113	1849	2875	1113	3348	1640	2201	1.040107138	7.644703084	50.25754122	1.35E-12	8.88E-10	-	3'UTR	Scn1b	protein_coding
7	127708549	127708749	211	359	239	378	259	363	1.044899377	4.80916637	30.80660798	2.85E-08	3.91E-06	-	5'UTR	Bcl7c	protein_coding
7	127708549	127708749	211	359	239	378	259	363	1.044899377	4.80916637	30.80660798	2.85E-08	3.91E-06	+	Exon	Mir762	miRNA
1	182019779	182019997	418	577	339	636	267	803	1.050387688	5.537638794	17.48362266	2.90E-05	0.000881328	-	5'UTR	Enah	protein_coding
9	101074113	101074337	375	534	187	500	243	598	1.064797178	5.226011788	17.9264055	2.30E-05	0.000753504	+	5'UTR	Msl2	protein_coding
5	5380352	5380552	121	179	85	212	117	173	1.108562521	3.76261992	39.73146447	2.91E-10	9.00E-08	-	Intron	Cdk14	protein_coding
8	46152183	46152396	236	403	147	384	221	373	1.10877178	4.76821243	34.39120393	4.51E-09	1.01E-06	+	Intron	Cfap97	protein_coding
11	3648544	3648751	226	521	171	468	225	429	1.10999125	4.967651945	36.85755231	1.27E-09	3.59E-07	-	Exon	Tug1	processed_transcript
4	117883230	117883445	652	1099	393	1076	545	1068	1.111408883	6.223589157	32.39591246	1.26E-08	1.94E-06	-	5'UTR	B4galt2	protein_coding
1	75553503	75553703	209	338	90	333	182	269	1.119131829	4.448688191	31.40380754	2.10E-08	3.14E-06	+	Exon	Slc4a3	protein_coding
1	75553503	75553703	209	338	90	333	182	269	1.119131829	4.448688191	31.40380754	2.10E-08	3.14E-06	+	3'UTR	Slc4a3	protein_coding
2	34406860	34407060	206	254	75	302	155	235	1.130589069	4.231949963	24.62177511	6.98E-07	5.26E-05	+	5'UTR	Mapkap1	protein_coding
12	55205544	55205744	17	45	11	46	19	26	1.172311674	1.343836497	17.80006218	2.45E-05	0.000781592	+	Exon	1700047I17Rik2	protein_coding
17	80954751	80954951	37	26	69	85	36	54	1.187259087	2.200115467	27.45181144	1.61E-07	1.64E-05	+	Intron	Tmem178	protein_coding
19	5272771	5272971	65	168	48	123	42	188	1.194210764	3.271737336	12.7403449	0.000357852	0.005435936	-	Exon	Pacs1	protein_coding
19	5272771	5272971	65	168	48	123	42	188	1.194210764	3.271737336	12.7403449	0.000357852	0.005435936	-	Start codon	Pacs1	protein_coding
19	5272771	5272971	65	168	48	123	42	188	1.194210764	3.271737336	12.7403449	0.000357852	0.005435936	-	5'UTR	Pacs1	protein_coding
11	106920532	106920732	124	283	76	281	135	201	1.194282687	4.072574397	50.9107049	9.67E-13	7.35E-10	-	5'UTR	Smurf2	protein_coding
11	106920532	106920732	124	283	76	281	135	201	1.194282687	4.072574397	50.9107049	9.67E-13	7.35E-10	-	Exon	Gm27322	miRNA
3	88024249	88024449	161	242	109	402	178	169	1.230001002	4.262177564	44.35451612	2.74E-11	1.13E-08	-	Exon	Hapln2	protein_coding
3	88024249	88024449	161	242	109	402	178	169	1.230001002	4.262177564	44.35451612	2.74E-11	1.13E-08	-	Start codon	Hapln2	protein_coding
3	88024249	88024449	161	242	109	402	178	169	1.230001002	4.262177564	44.35451612	2.74E-11	1.13E-08	-	5'UTR	Hapln2	protein_coding
1	164328841	164329041	51	36	76	101	43	87	1.243650918	2.566152832	35.45310097	2.61E-09	6.30E-07	+	Intron	Nme7	protein_coding
8	14307057	14307278	261	302	307	680	298	429	1.338734253	5.127607967	81.25582918	1.98E-19	1.96E-16	+	Intron	Dlgap2	protein_coding
8	14306798	14307003	609	752	640	1531	699	1094	1.387398293	6.373486736	101.6481041	6.63E-24	8.19E-21	+	Intron	Dlgap2	protein_coding
13	36206170	36206370	72	52	70	188	49	98	1.398749653	2.978877618	40.28056312	2.20E-10	7.01E-08	+	Intron	Fars2	protein_coding
5	84066640	84066840	146	75	101	288	104	156	1.414227626	3.711707075	44.94744048	2.02E-11	8.70E-09	-	3'UTR	Epha5	protein_coding
7	75252911	75253111	22	30	80	148	19	70	1.476543489	2.440634144	14.19617673	0.000164705	0.003112701	-	Intron	Sv2b	protein_coding
2	29125101	29125301	195	373	47	308	197	366	1.531757824	4.55241228	24.76477072	6.48E-07	4.96E-05	+	5'UTR	Setx	protein_coding
15	83779708	83779908	88	119	28	193	80	132	1.544402116	3.297930924	46.75253295	8.05E-12	4.19E-09	+	Intron	Mpped1	protein_coding
11	87262974	87263174	42	61	61	151	85	66	1.544761196	2.84202525	46.01443402	1.17E-11	5.80E-09	-	Intron	Ppm1e	protein_coding
Y	90814153	90814353	20	22	11	57	22	43	1.863586875	1.45087595	47.39700257	5.80E-12	3.18E-09	+	Intron	Erdr1	protein_coding
Y	90811325	90811563	300	297	169	781	384	610	1.892293417	5.349129008	103.7992448	2.24E-24	3.16E-21	+	Intron	Erdr1	protein_coding
Y	90811811	90812033	241	189	182	749	276	528	1.970258246	5.090685395	118.365884	1.44E-27	2.38E-24	+	Intron	Erdr1	protein_coding
Y	90812185	90812431	255	244	240	873	384	619	1.993298515	5.383064267	182.3027699	1.52E-41	7.53E-38	+	Intron	Erdr1	protein_coding
Y	90812486	90812686	104	106	73	400	211	392	2.511618746	4.398334623	159.4137949	1.52E-36	3.75E-33	+	Intron	Erdr1	protein_coding
13	8330619	8330819	61	32	39	263	94	198	2.719730836	3.444634434	147.9231414	4.93E-34	9.75E-31	+	Intron	Adarb2	protein_coding
