# Supplementary material for: The N6‐methyladenosine RNA landscape in the aged mouse hippocampus
Source: Aging Cell. 2022 Dec 9;22(1):e13755. doi: 10.1111/acel.13755 (PMC9835576; doi:10.1111/acel.13755)
Supplement: Supplementary file 3 — Table S2. KEGG pathways associated with differentially methylated genes [file ACEL-22-e13755-s004.pdf]

ID	Description	GeneRatio	BgRatio	pvalue	p.adjust	qvalue	geneID	Count
mmu04728	Dopaminergic synapse	12/147	135/9000	1.90E-06	0.000425	0.000336	Cacna1a/Gnai2/Gnao1/Adcy5/Grin2a/Plcb3/Ppp2r5a/Ppp2cb/Gsk3a/Cacna1b/Creb3/Kif5c	12
mmu04724	Glutamatergic synapse	10/147	113/9000	1.47E-05	0.001096	0.000865	Adcy2/Shank3/Cacna1a/Shank1/Gnai2/Gls/Gnao1/Adcy5/Grin2a/Plcb3	10
mmu04727	GABAergic synapse	9/147	89/9000	1.35E-05	0.001096	0.000865	Adcy2/Cacna1a/Gnai2/Gls/Gnao1/Adcy5/Src/Gabbr1/Cacna1b	9
mmu04927	Cortisol synthesis and secretion	8/147	72/9000	2.07E-05	0.00116	0.000916	Adcy2/Pde8b/Nr4a1/Adcy5/Kcnk2/Plcb3/Kcnk3/Creb3	8
mmu04015	Rap1 signaling pathway	13/147	214/9000	4.53E-05	0.001879	0.001483	Adcy2/Gnai2/Enah/Gnao1/Adcy5/Magi3/Grin2a/Plcb3/Magi2/Fgf20/Src/Cnr1/Tln1	13
mmu04730	Long-term depression	7/147	60/9000	5.03E-05	0.001879	0.001483	Cacna1a/Gnai2/Gnao1/Plcb3/Ryr1/Ppp2cb/Gna12	7
mmu04725	Cholinergic synapse	9/147	112/9000	8.52E-05	0.002726	0.002152	Adcy2/Kcnq3/Cacna1a/Gnai2/Gnao1/Adcy5/Plcb3/Cacna1b/Creb3	9
mmu04022	cGMP-PKG signaling pathway	11/147	173/9000	0.000119	0.002955	0.002333	Adcy2/Gnai2/Adcy5/Kcnmb4/Myh7/Irs2/Kcnma1/Plcb3/Atp1a2/Creb3/Gna12	11
mmu05032	Morphine addiction	8/147	91/9000	0.000113	0.002955	0.002333	Adcy2/Cacna1a/Gnai2/Pde8b/Gnao1/Adcy5/Gabbr1/Cacna1b	8
mmu04261	Adrenergic signaling in cardiomyocytes	10/147	152/9000	0.000184	0.004131	0.003261	Adcy2/Gnai2/Adcy5/Myh7/Plcb3/Ppp2r5a/Ppp2cb/Atp1a2/Creb3/Scn1b	10
mmu04934	Cushing syndrome	10/147	162/9000	0.000309	0.00623	0.004918	Adcy2/Gnai2/Kmt2d/Pde8b/Nr4a1/Adcy5/Kcnk2/Plcb3/Kcnk3/Creb3	10
mmu04915	Estrogen signaling pathway	9/147	134/9000	0.000334	0.00623	0.004918	Adcy2/Gnai2/Krt9/Gnao1/Adcy5/Plcb3/Src/Gabbr1/Creb3	9
mmu04144	Endocytosis	13/147	272/9000	0.000493	0.007896	0.006234	Rab11fip2/Snx3/Agap2/Arf3/Psd/Rab10/Rab11fip5/Iqsec2/Agap1/Smap2/Src/Smurf2/Kif5c	13
mmu04911	Insulin secretion	7/147	86/9000	0.000492	0.007896	0.006234	Adcy2/Adcy5/Kcnmb4/Kcnma1/Plcb3/Atp1a2/Creb3	7
mmu04935	Growth hormone synthesis, secretion and action	8/147	117/9000	0.000637	0.009506	0.007505	Adcy2/Gnai2/Adcy5/Irs2/Plcb3/Ep300/Sstr3/Creb3	8
mmu04611	Platelet activation	8/147	125/9000	0.000984	0.013778	0.010877	Adcy2/Gnai2/Arhgap35/Arhgef12/Adcy5/Plcb3/Src/Tln1	8
mmu04713	Circadian entrainment	7/147	98/9000	0.001076	0.014181	0.011196	Adcy2/Gnai2/Gnao1/Adcy5/Grin2a/Plcb3/Ryr1	7
mmu04916	Melanogenesis	7/147	100/9000	0.001212	0.015084	0.011908	Adcy2/Gnai2/Gnao1/Adcy5/Plcb3/Ep300/Creb3	7
mmu04971	Gastric acid secretion	6/147	75/9000	0.001367	0.015309	0.012086	Adcy2/Gnai2/Adcy5/Kcnk2/Plcb3/Atp1a2	6
mmu04925	Aldosterone synthesis and secretion	7/147	102/9000	0.001361	0.015309	0.012086	Adcy2/Nr4a1/Adcy5/Plcb3/Kcnk3/Atp1a2/Creb3	7
mmu04928	Parathyroid hormone synthesis, secretion and action	7/147	108/9000	0.001895	0.020213	0.015958	Adcy2/Gnai2/Adcy5/Plcb3/Jund/Creb3/Gna12	7
mmu04270	Vascular smooth muscle contraction	8/147	144/9000	0.002429	0.02473	0.019524	Adcy2/Myh14/Arhgef12/Adcy5/Kcnmb4/Kcnma1/Plcb3/Gna12	8
mmu04723	Retrograde endocannabinoid signaling	8/147	148/9000	0.002879	0.026873	0.021216	Adcy2/Cacna1a/Gnai2/Gnao1/Adcy5/Plcb3/Cnr1/Cacna1b	8
mmu04360	Axon guidance	9/147	181/9000	0.002813	0.026873	0.021216	Gnai2/Enah/Sema7a/Arhgef12/Unc5b/Epha5/Src/Efnb3/Pak6	9
mmu04921	Oxytocin signaling pathway	8/147	153/9000	0.003531	0.031641	0.024979	Adcy2/Gnai2/Gnao1/Adcy5/Plcb3/Ryr1/Src/Eef2k	8
mmu00532	Glycosaminoglycan biosynthesis - chondroitin sulfate / dermatan sulfate	3/147	20/9000	0.003969	0.034198	0.026998	Xylt1/Chst11/Chpf	3
mmu04926	Relaxin signaling pathway	7/147	129/9000	0.005119	0.042468	0.033527	Adcy2/Gnai2/Gnao1/Adcy5/Plcb3/Src/Creb3	7
