# Supplementary material for: The N6‐methyladenosine RNA landscape in the aged mouse hippocampus
Source: Aging Cell. 2022 Dec 9;22(1):e13755. doi: 10.1111/acel.13755 (PMC9835576; doi:10.1111/acel.13755)
Supplement: Supplementary file 4 — Figure S1. Validation of aging‐related gene expression changes in the hippocampus [file ACEL-22-e13755-s003.pdf]

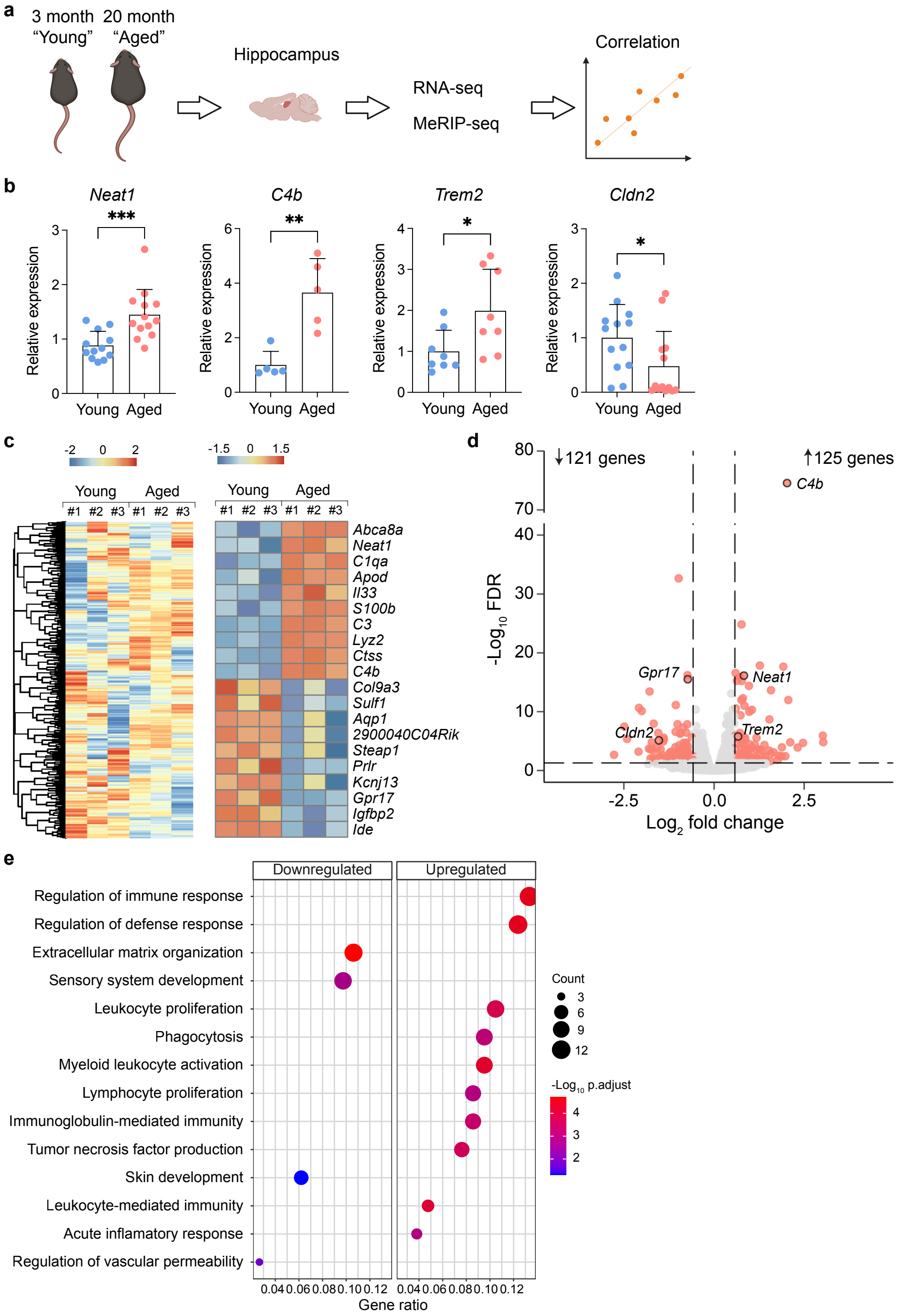

Huang et al. - Supplementary Figure 1

sFigure 1. Validation of aging-related gene expression changes in the hippocampus.

(a). The study design using C57BL/6 mice of 3 months and 20 months of age. The isolated hippocampal RNA was subjected to RNA-seq and MeRIP-seq analyses.
(b). qRT-PCR validation of aging-related expression changes in Neat1, C4b, Trem2, and Cldn2 transcript levels. Rpl13a was used as a normalization gene. Data are presented as mean ± SD (Mann-Whitney U test, $^{*}P < 0.05$, $^{**}P < 0.01$, $^{***}P < 0.001$).
(c). Heatmap summary of all RNA-seq read counts. The top 10 downregulated and upregulated genes in the aged hippocampus are shown on the right.
(d). Volcano plot showing differentially expressed genes (DEGs) in the aged relative to the young hippocampus using fold-change of 1.5 and an FDR of 0.05 as threshold.
(e). GO enrichment analyses of the age-induced DEGs.
