# Supplementary material for: The N6‐methyladenosine RNA landscape in the aged mouse hippocampus
Source: Aging Cell. 2022 Dec 9;22(1):e13755. doi: 10.1111/acel.13755 (PMC9835576; doi:10.1111/acel.13755)
Supplement: Supplementary file 5 — Figure S2. m6A peaks in young and aged animals [file ACEL-22-e13755-s001.pdf]

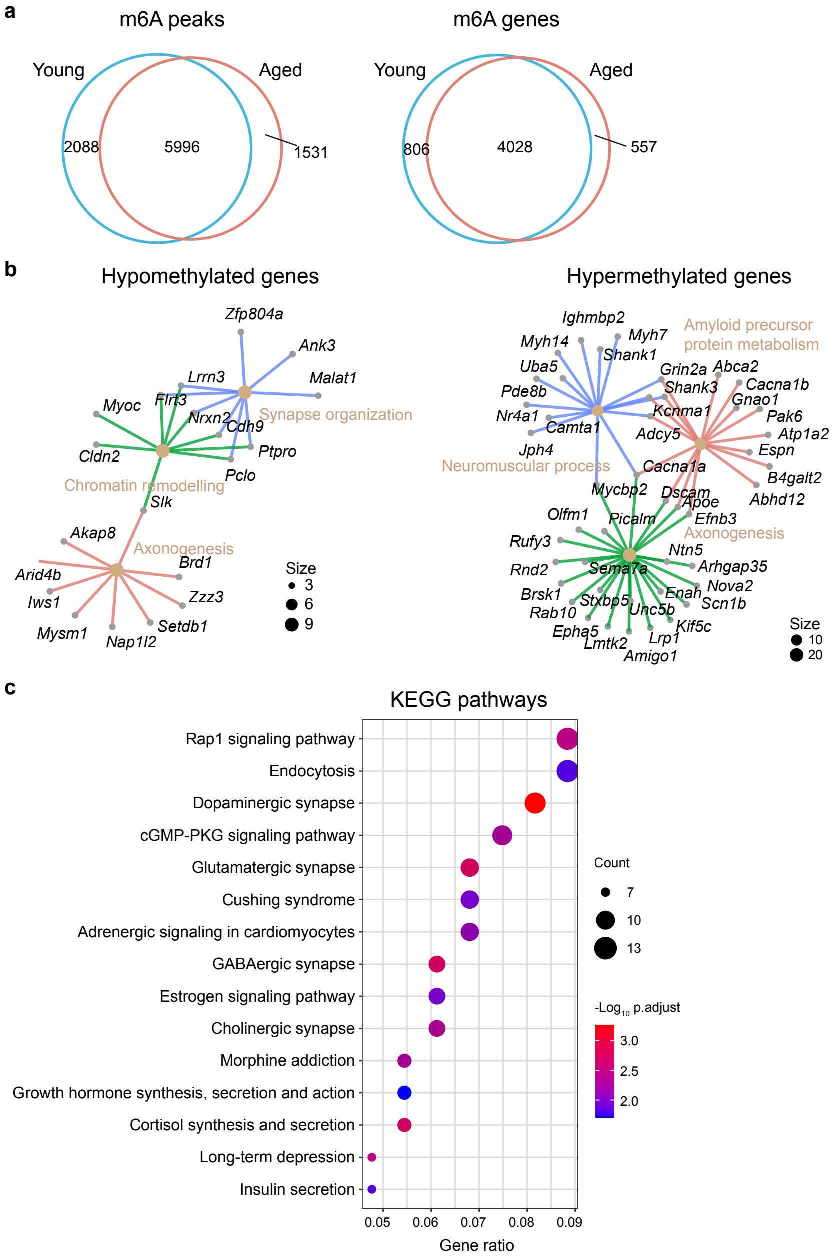

Huang et al. - Supplementary Figure 2

sFigure 2. MeRIP-seq associated peaks in young and aged animals
(a). Venn diagram showing the total number of peaks and genes identified in young and aged mouse brain.
(b). Gene-Concept Network analysis displaying the three most significantly enriched GO terms in hypomethylated genes (left) and hypermethylated genes (right).
(c). KEGG pathway analyses of modulated m6A peaks that are modulated with age.
